# Cytotoxic activity of IMMUNEPOTENT CRP against non-small cell lung cancer cell lines

**DOI:** 10.7717/peerj.7759

**Published:** 2019-09-27

**Authors:** Ana Carolina Martinez-Torres, Luis Gomez-Morales, Alan B. Martinez-Loria, Ashanti Concepcion Uscanga-Palomeque, Jose Manuel Vazquez-Guillen, Cristina Rodriguez-Padilla

**Affiliations:** Facultad de Ciencias Biológicas, Laboratorio de Inmunología y Virología, Universidad Autónoma de Nuevo León, San Nicolás de los Garza, Nuevo León, Mexico

**Keywords:** Transfer factor, Non small cell lung cancer, NSCLC, ROS, Cell cycle, Cell death, Apoptosis, Reactive oxygen species, Mitochondrial damage

## Abstract

**Background:**

IMMUNEPOTENT-CRP® (I-CRP) is a bovine dialyzable leukocyte extract containing transfer factor. It is a cost-effective, unspecific active immunotherapy that has been used in patients with non-small cell lung cancer (NSCLC) as an adjuvant to reduce the side-effects of chemotherapy and radiotherapy, and has shown cytotoxic activity *in vitro* on different cancer cell lines. However, its mechanism of action against lung cancer cells has not been assessed. Therefore, the objective of this work was to assess the cytotoxic mechanism of I-CRP on lung cancer cell lines.

**Methods:**

We assessed cell viability through MTT assay on the NSCLC cell lines A549, A427, Calu-1, and INER-51 after treatment with I-CRP. To further understand the mechanisms of cell viability diminution we used fluorescence-activated cell sorting to evaluate cell death (annexin-V and propidium iodide [PI] staining), cell cycle and DNA degradation (PI staining), mitochondrial alterations (TMRE staining), and reactive oxygen species (ROS) production (DCFDA staining). Additionally, we evaluated caspase and ROS dependence of cell death by pretreating the cells with the pan-caspase inhibitor Q-VD-OPH and the antioxidant N-acetylcysteine (NAC), respectively.

**Results:**

Our data shows that I-CRP is cytotoxic to NSCLC cell lines in a dose and time dependent manner, without substantial differences between the four cell lines tested (A549, A427, Calu-1, and INER-51). Cytotoxicity is induced through regulated cell death and cell cycle arrest induction. I-CRP-induced cell death in NSCLC cell lines is characterized by DNA degradation, mitochondrial damage, and ROS production. Moreover, cell death is independent of caspases but relies on ROS production, as it is abrogated with NAC.

**Conclusion:**

Altogether, these results improve the knowledge about the cytotoxic activity of I-CRP on NSCLC cells, indicating that cell death, cell cycle arrest, DNA degradation and mitochondrial damage are important features, while ROS play the main role for I-CRP mediated cytotoxicity in lung cancer cells.

## Introduction

Lung cancer is the leading cause of cancer death in men and the second in women worldwide (Torre et al., 2016; Bray et al., 2018). Non-small cell lung cancer (NSCLC) is the most common type of lung cancer, accounting for ∼85% of cases (Zappa & Mousa, 2016). Despite the intensive research on radiotherapy and chemotherapy over the past few decades, prognosis for patients with NSCLC has not improved significantly when compared to other types of cancer, such as breast cancer (Sundquist, Brudin & Tejler, 2017), being the overall 5-year free survival rate of 18% (Pastorino, 2010; Torre et al., 2016). Given the ineffective anti-tumor treatments or their adverse side effects, many efforts have been made to find more effective and safe therapies than conventional drugs and, recently, active immunotherapies based on immune checkpoint blockade monoclonal antibodies have demonstrated to harvest favorable results (Massarelli et al., 2014; Chodon, Koya & Odunsi, 2015).

Dialyzable leukocyte extracts (DLEs) are biological preparations containing different low-molecular-weight substances with immunomodulatory properties that are usually referred to as transfer factor. DLEs are cost-effective, non-specific, active immunotherapies with protective and therapeutic effects in cancer and different infectious and allergic diseases (Arnaudov & Kostova, 2015). IMMUNEPOTENT CRP (I-CRP) is a DLE obtained from bovine spleen, with immunomodulatory but also cancer-cytotoxic activity. I-CRP has shown to reduce viability of different types of cancer cell lines but not peripheral blood mononuclear cells (PBMCs) (Franco-Molina et al., 2006). It induces DNA fragmentation and suppression of *p53*, *bag-1*, *c-myc*, *bax*, *bcl-2*, and *bad* mRNA expression in the MCF-7 breast cancer cell line (Franco-Molina et al., 2006). Conversely, I-CRP protects mice against 5-Fluorouracil-induced bone marrow myeloablation (Coronado-Cerda et al., 2016), while it increases the rate of oxaliplatin-induced immunogenic cell death in murine melanoma cells (Rodríguez-Salazar et al., 2017). This emphasizes its broad, yet misunderstood, specific cytotoxicity to cancer cells.

Recently, we described cellular and biochemical changes in HeLa cells after I-CRP treatment and found reactive oxygen species (ROS) production but not caspase activation to be responsible for its cytotoxicity (Martínez-Torres et al., 2018a). However, evidence in cancer types in which the use of I-CRP has already been described as clinically relevant, such as lung cancer, has not been reported yet. Indeed, clinical assessment of I-CRP in patients with breast and lung cancer undergoing standard chemotherapy regimens has shown to improve overall quality of patients’ lives due to its adjuvant capacity in breast (Lara et al., 2010) and lung cancer patients (Franco-Molina et al., 2008). Nevertheless, understanding the cytotoxic mechanism of I-CRP in these types of cancer might lead to the improvement and optimization of its use, and combination with chemotherapies in cancer patients.

For all the above, the aim of the present study was to investigate whether I-CRP was cytotoxic in NSCLC cells, and to elucidate the mechanism of its cytotoxicity. Therefore, we assessed cell viability, cell death, cell cycle, and mitochondrial damage after I-CRP-treatment. Furthermore, we evaluated the role of caspases and ROS production in its cytotoxic effect.

## Materials and Methods

### Cell culture

A549 (ATCC® CCL-185™), A427 (ATCC® HTB-53™), and Calu-1 (ATCC® HTB-54™) cell lines were obtained from the ATCC (Manassas, VA, USA), INER-51 (CVCL_5531) was a kind gift from the National Institute of Respiratory Diseases (Mexico). The cells were incubated in a humidified atmosphere with 5% CO_2_ at 37 °C and were maintained in culture medium DMEM/F-12 containing 2.50 mM L-Glutamine, 15 mM HEPES buffer medium (Gibco, Grand Island NY, USA) supplemented with 10% heat-inactivated fetal bovine serum (FBS) (Gibco, Grand Island NY, USA) and 100 U/mL penicillin/streptomycin (Gibco, Grand Island NY, USA). The cell lines were grown in plastic tissue-culture dishes (Corning, NY, USA).

### Cell death induction and inhibition

The bovine dialyzable leukocyte extract, IMMUNEPOTENT CRP (I-CRP) was produced by the Laboratorio de Inmunología y Virología at the Facultad de Ciencias Biológicas of the Universidad Autónoma de Nuevo León (San Nicolás de los Garza, Nuevo León, México) and was dissolved in cell culture medium. One unit of I-CRP is defined as the product obtained from 1 × 10^8^ leukocytes (Arnaudov & Kostova, 2015). Etoposide (Milliporesigma, St. Louis, MO, USA) and Q-VD.OPh (QVD) (BioVision) were dissolved in DMSO (Milliporesigma, St. Louis, MO, USA). N-acetyl-L-cysteine (NAC) (Milliporesigma, St. Louis, MO, USA) was dissolved in MiliQ water. For cell death inhibition we used QVD, as caspase inhibitor, and we used NAC as a ROS scavenger. QVD, and NAC were added 30 min before I-CRP treatment. All stock solutions were wrapped in foil and stored at −20 °C.

### Cell viability assessment

Cell growth inhibition was determined by measuring formazan absorbance after 3-(4,5-dimethylthiazol-2-yl)-2,5-diphenyltetrazolium bromide (MTT) reduction by living cells. In brief, 1 × 10^4^ cells per well were seeded in 96-well plates (Corning, NY, USA) for MTT assays. After exposure to I-CRP at growing concentrations (0.25–2 U/mL in 100 µL as final volume) for 24, 48, and 72 h, 20 µL of MTT solution (2 mg/mL in PBS) were added to each well. The plates were incubated for three additional hours at 37 °C, after which the MTT solution in the medium was aspirated and 200 µL of DMSO were added to each well to solubilize the formazan crystals formed in the viable cells. The optical density was measured at 570 nm using a Biotek Synergy2 microplate reader (Winooski, VT, USA). Experiments were done at least two times in triplicates.

### Clonogenic assay

Cell clonogenicity was assessed as described previously (Martínez-Torres et al., 2018b). Briefly, in 6-well plates, 100 cells were seeded to use as control, and 500 cells for treatment, and were incubated overnight. Once attached, cells were exposed to treatment or culture media alone for 24 h. Afterward, the medium was replaced, and cells were allowed to grow until colony formation (10 days). Then, the colonies were fixed with methanol and glacial acetic acid (3:1), stained with 0.5% crystal violet (SIGMA), washed with PBS and colonies with >50 cells were counted manually. Cell survival was determined by equating one fifth of the number of colonies in treated wells over those in control.

### Cell death analysis

Cell death was determined by staining cells with Annexin-V-APC staining (AnnV) (BD Biosciences Pharmigen, San Jose, CA, USA) and propidium iodide (PI) (Milliporesigma, St. Louis, MO, USA). 4 × 10^4^ cells were seeded in 24-well plates (Corning, NY, USA) and were incubated at different concentrations of I-CRP (1.25–1.75 U/mL in a final volume of 400 µL) for 24 h to find the cytotoxic concentration 50 (CC_50_). After 24 h, the cells were detached and washed with PBS, then resuspended in 200 µL of Annexin-V binding buffer (10 mM HEPES/NaOH pH 7.4, 140 mM NaCl, 2.5 mM CaCl_2_) containing 0.1 µg/mL AnnV and 0.5 µg/mL PI, and incubated at 4 °C for 20 min. Fluorescence-activated sorting of 10^4^ cells was then assessed with a BDAccuri C6 flow cytometer (Becton Dickenson, San Jose, CA, USA) and analyzed using FlowJo Software V.10 (Tree Star Inc, Ashland, OR, USA).

### Cell cycle analysis and DNA degradation

Cell cycle distributions were determined by PI staining. In brief, 2 × 10^5^ cells in 2 mL were seeded in 6-well dishes (Corning, NY, USA) and incubated with I-CRP, CC_50_, for 24, 48 or 72 h. Cells were then washed with PBS and fixed overnight at −20 °C in 70% ethanol. Cells were washed again with PBS, then incubated with PI (10 µg/mL; Milliporesigma, St. Louis, MO, USA) with simultaneous RNAse (Milliporesigma, St. Louis, MO, USA) treatment at 37 °C for 30 min. DNA contents of 10^4^ cells were measured using a flow cytometer (Becton Dickinson, BDAccuri C6), and analyzed using FlowJo Software V.10 (Tree Star Inc, Ashland, OR, USA). For DNA degradation, we analyzed the Sub-G1 population obtained from cell cycle analysis using a BDAccuri C6 flow cytometer (Becton Dickenson, San Jose, CA, USA), and analyzed using FlowJo Software V.10 (Tree Star Inc, Ashland, OR, USA).

### Mitochondrial membrane potential (Δψm) analysis

Loss of Δψm was measured using TMRE (tetramethylrhodamine, ethyl ester) (Molecular Probes, OR, USA) a cationic and cell permeable fluorescent dye. 4 × 10^4^ cells were seeded in 24-well dishes (Corning, NY, USA), and incubated with CC_50_ I-CRP. Cells were then recovered, washed with PBS, stained with 50 nM TMRE (dissolved in PBS), incubated at 37 °C for 30 min, washed again, and suspended in PBS. TMRE fluorescence in 10^4^ cells was measured using a BDAccuri C6 flow cytometer (Becton Dickenson, San Jose, CA, USA) and analyzed using FlowJo Software V.10 (Tree Star Inc, Ashland, OR, USA).

### ROS production assay

ROS generation was measured using 2.5 µM DCFDA (dichlorodihydrofluorescein diacetate; Thermo Fisher Scientific, Waltham, MA, USA). In brief, 4 × 10^4^ cells in 24-well dishes (Corning, NY, USA) were left untreated or incubated with CC_50_ I-CRP, 5 mM NAC (Milliporesigma, St. Louis, MO, USA), or 5 mM NAC + CC_50_ I-CRP for 24 h. Cells were then recovered, washed with PBS, stained, incubated at 37 °C for 30 min, and 10^4^ cells were fluorescence-sorted using a BDAccuri C6 flow cytometer (Becton Dickenson, San Jose, CA, USA). Finally, data was analyzed using FlowJo Software V.10 (Tree Star Inc, Ashland, OR, USA).

### Statistical analysis

The results given in this study represent the mean of at least three independent experiments done in triplicate (mean ± SD), unless specified. Statistical analysis was done using paired student T-tests, and statistical significance was defined as *p* < 0.05. The data was analyzed using GraphPad Prism 6.0 (San Diego, CA, USA).

## Results

### IMMUNEPOTENT CRP® (I-CRP) inhibits cell viability in non-small cell lung cancer (NSCLC) cell lines

**Figure 1:**
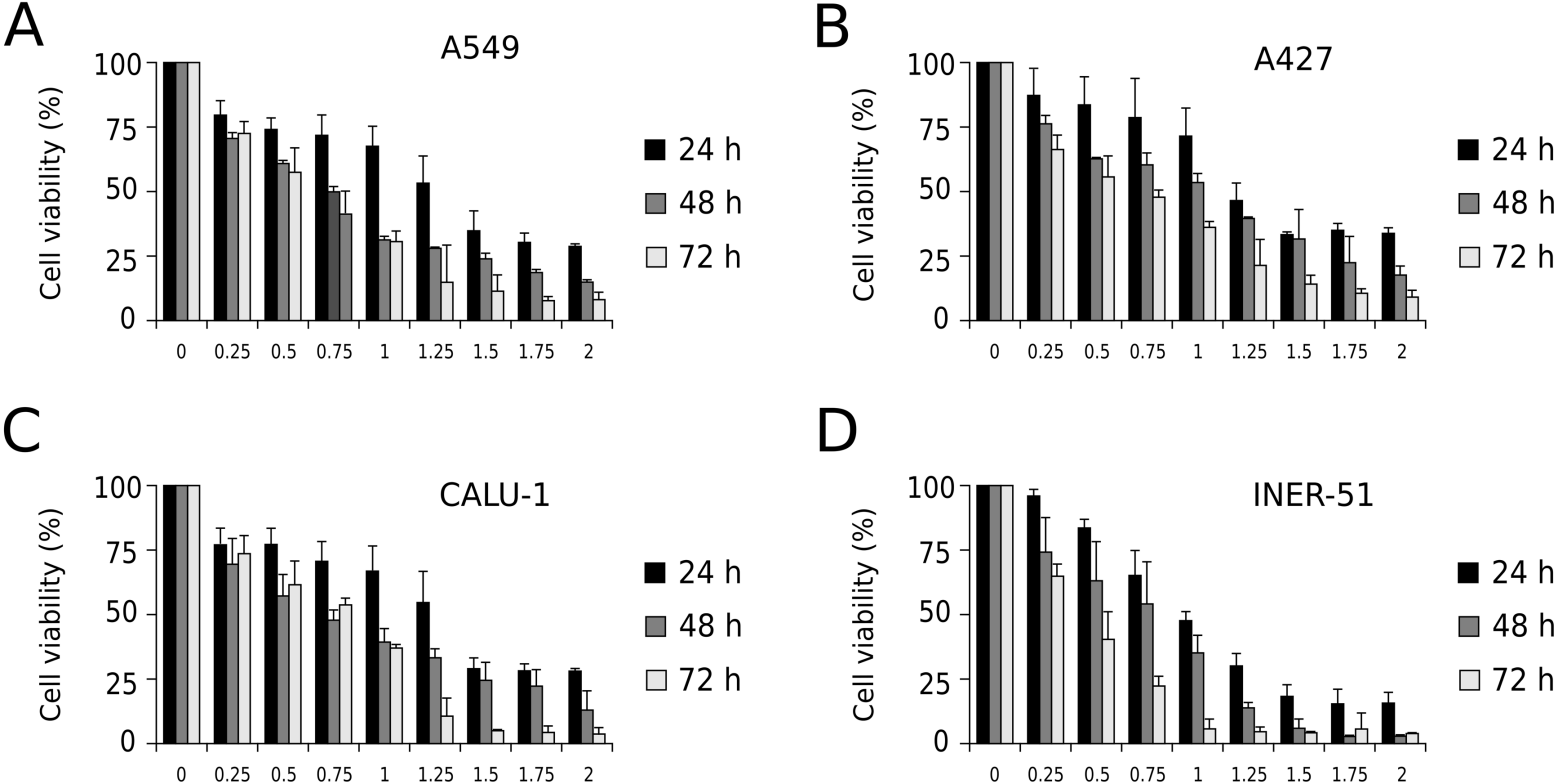
I-CRP reduces viability of NSCLC cell lines in a time- and dose-dependent manner. MTT assays were performed in A549 (A), A427 (B), CALU-1 (C), and INER-51 (D) cells. Viability of cells treated with increasing I-CRP concentrations (0, 0.25, 0.5, 0.75, 1.0, 1.25, 1.5, 1.75 and 2.0 U/mL) for 24, 48, and 72 h were obtained by normalizing the absorbance measured in each case over those of its respective untreated control (0 U/mL). Bar graphs represent the means (±SD) of triplicates of three independent studies.

First, to evaluate I-CRP cytotoxicity in non-small cell lung cancer (NSCLC) cell lines, we assessed indirect cell viability through MTT assay in A549, A427, CALU-1, and INER-51 lung carcinoma cells. We treated the cells with 0.25–2 U/mL of I-CRP during 24, 48, and 72 h and observed concentration-dependent cell viability diminution in a time-dependent manner for the four cell lines tested (Fig. 1). Overall, the different cell lines tested conserved a similar sensitivity to I-CRP. In fact, the concentration of I-CRP that diminished the 50% of cell viability in all the cell lines tested was of 1.0–1.25 U/mL after 24 h of treatment, of 0.75–1.0 U/mL after 48 h of treatment, and of 0.5–0.75 U/mL after 72 h of treatment (Table 1). These results indicate that I-CRP induces similar cytotoxicity to different lung cancer cell lines in a time- and concentration-dependent manner.

### Cell death and cell cycle arrest in NSCLC cell lines after treatment with I-CRP

Since MTT is an indirect assay that evaluates the activity of mitochondrial dehydrogenases, we decided to delve into the single-cell effects involved in the cell viability loss caused by the I-CRP. As similar cytotoxic effects were observed in the four cell lines (Table 1) we chose for further assays two representative cell lines, A549 and A427 cell lines. These are two widely-used models of NSCLC with proved genetic differences and drug sensitivity (Giard et al., 1973; Lu & Arthur, 1992; Berger et al., 1999; Kutkowska, Strzadala & Rapak, 2017).

**Figure 2:**
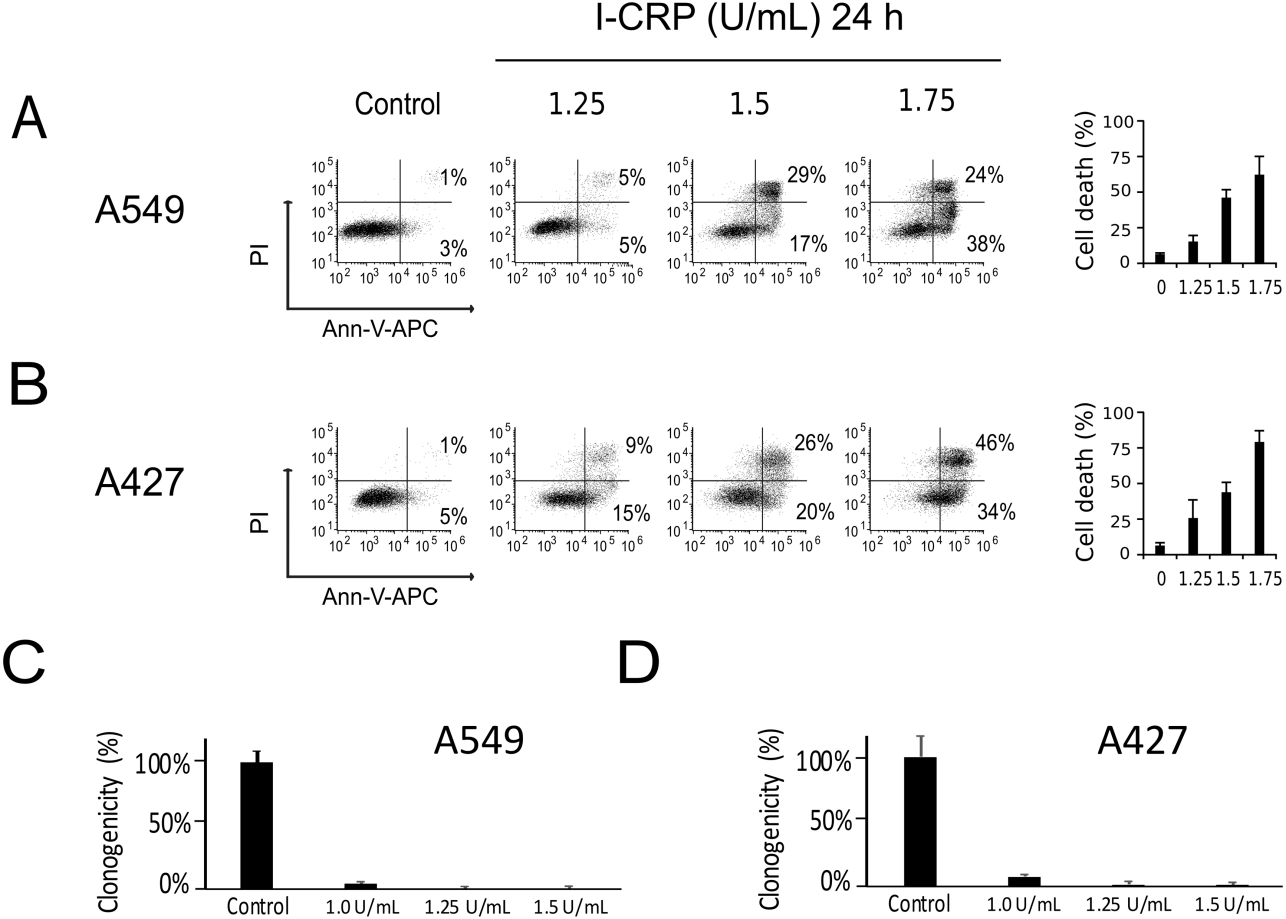
I-CRP kills A549 and A427 NSCLC cell lines. Phosphatidylserine exposure and plasma membrane permeability in A549 (A) and A427 (B) cells were measured by flow cytometry through the annexin-V-APC (Ann-V-APC) and propidium iodide (PI) staining. Cells were left alone (Control) or treated with increasing I-CRP concentrations (1.25, 1.5, 2 U/mL) for 24 h. Percentages indicate the number of cells with Ann-V-APC^+^/PI^+^ and Ann-V-APC^+^/PI^−^ staining analyzed by Flowjo software V.10. The sum of both quadrants were gathered together, and the means (±SD) of triplicates of three independent experiments are represented in bar graphs at the right. A549 (C) and A427 (D) cells were treated with different concentrations of I-CRP for 24 hours, the number of colonies formed on the culture plate 10 days after treatment was expressed as surviving fractions. Data are reported as the means (±SD) of the percentage of colonies compared with untreated control.

We performed a cell-death analysis through the evaluation of phosphatidylserine exposure and plasma membrane permeability using AnnV/PI staining. We found that I-CRP effectively induces cell death in a concentration-dependent manner in A549 (Fig. 2A) and A427 (Fig. 2B) cells. Note that in both cases I-CRP CC_50_ remains at 1.5 U/mL.

**Table 1:** IC50 of ICRP in lung cancer cells. Summary of ICRP concentrations (U/mL) necessary to reduce 50% of cell viability (measured by MTT assays) in lung cancer cell lines.

	**24 h**	**48 h**	**72 h**
A549	1.25	0.75	0.25
A427	1.25	1.0	0.75
CALU-1	1.25	0.75–1.0	0.75
INER-51	1.0	0.75	0.25–0.5

Using the CC_50_ and two lower concentrations (1.25 and 1.0 U/mL), we performed colony formation assays and found that I-CRP significantly reduced the clonogenicity of A549 (Fig. 2C) and A427 (Fig. 2D) cells. In both cases, 1.25 U/mL (where I-CRP only induces around 10% and 25% cell death in A549 and A427 cells, respectively), provoked the complete loss of cell clonogenicity, while at the even lower concentration of 1.0 U/mL the same was reduced to 3% and 7% respectively.

**Figure 3:**
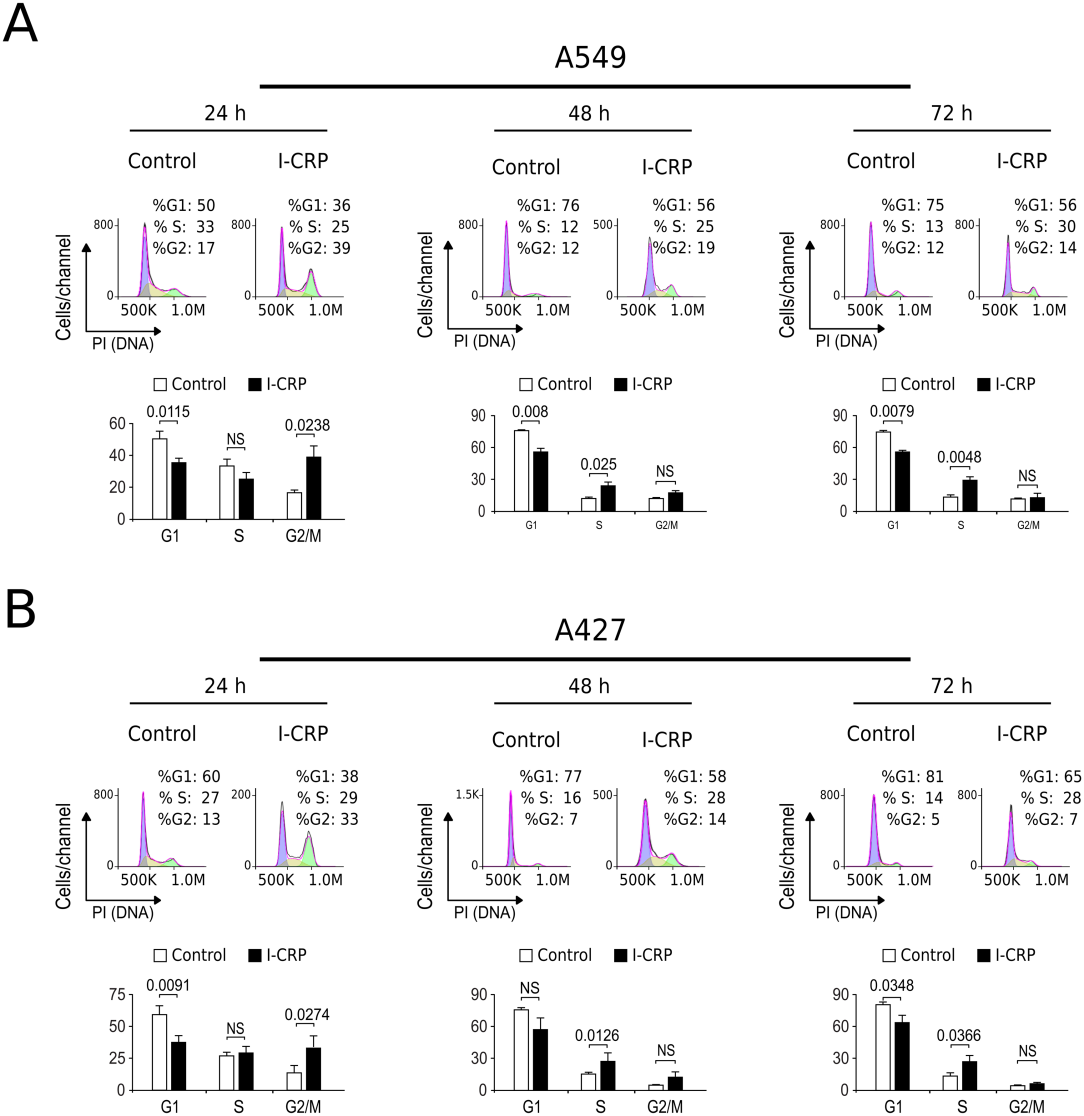
I-CRP induces cell cycle alterations in A549 and A427 cell lines. Cell cycle distributions (G1, S, and G2/M) in A549 (A) and A427 (B) cells left alone (Control) or treated with 1.5 U/mL I-CRP for 24, 48, and 72 h were calculated based on their DNA content, which was measured by flow cytometry through PI staining. Histograms at the upper side of each case are representative analyses of the experiments, performed using FlowJo software V.10. Bar graphs represent the means (±SD) of triplicates of three independent experiments. Statistical analyses were performed using *t*-tests.

Interestingly, we had observed that higher I-CRP concentrations were needed to kill both lung cancer cell lines than to make them lose their ability to form colonies or reduce MTT. Thus, we hypothesized that such discrepancies could be due to cell cycle arrest, as it was recently shown to occur in HeLa cells (Martínez-Torres et al., 2018a). Consequently, we proceeded to evaluate cell cycle in A549 and A427 cell lines after I-CRP treatment. Cell cycle analysis shows that I-CRP CC_50_ induces cell cycle arrest in G2/M after 24 h treatment in both, A549 (Fig. 3A) and A427 (Fig. 3B) cell lines. Moreover, after 48 h and 72 h, it also leads to differences in S or G2/M phase in both cell lines as well; however, in these cases an arrest cannot be inferred as the percentage of cells in G2/M and S in controls can diminish when cells are cultured in similar conditions for longer than 24 h (Friedman et al., 2013; Rahman et al., 2014; Wang et al., 2016). This indicates that I-CRP induces similar cell cycle arrest in A549 and A427 NSCLC cell lines, which in part explains the differences in the concentration of I-CRP needed to inhibit cell viability and the one needed to induce cell death.

### I-CRP treatment induces DNA degradation, mitochondrial damage, and caspase-independent cell death in NSCLC cells

**Figure 4:**
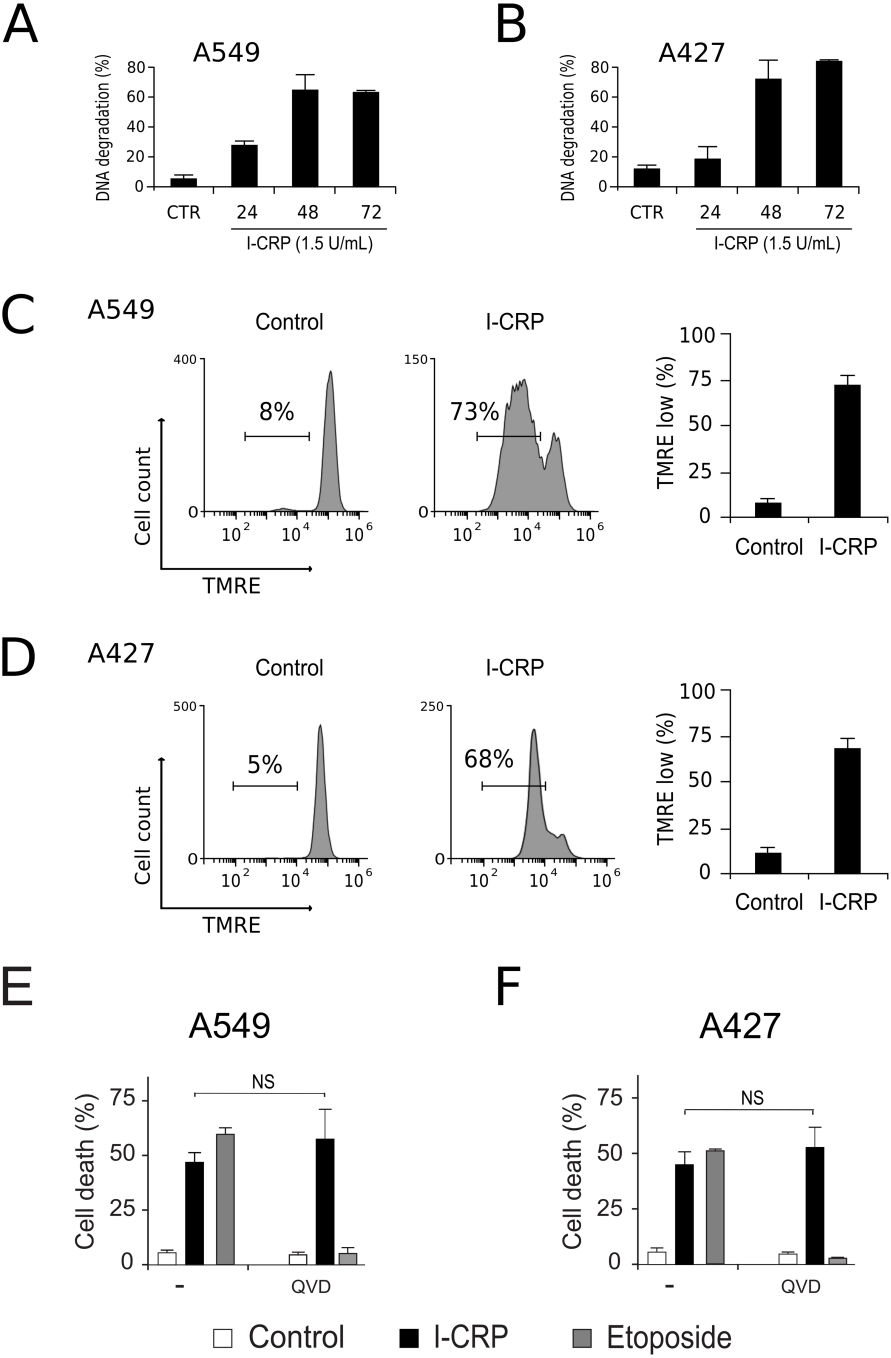
I-CRP induces DNA degradation, loss of mitochondrial membrane potential, and caspase-independent cell death in A549 and A427 cells. DNA degradation in A549 (A) or A427 (B) cells left alone (Control) or treated with I-CRP (1.5 U/mL) for 24, 48, and 72 h, was measured by flow cytometry through PI staining. Bar graphs represent the percentage if cells in the Sub-G1 phase of the cell cycle. (C–D) Mitochondrial membrane potential was measured by flow cytometry through TMRE staining in A549 (C) and A427 (D) cells left alone (Control) or treated with I-CRP (1.5 U/mL) for 24 h. The representative histograms indicate the percentage of cells with low TMRE fluorescence intensity. (E–F) Cell death of A549 (E) and A427 (F) cells was measured by flow cytometry through the Ann-V-APC/PI staining of cells left alone (Control) and cells treated with I-CRP (1.5 U/mL) or ETO (200 µM) for 24 h in presence or absence (−) of pan-caspase inhibitor Q-VD-OPH (QVD). Statistical analysis was performed using *t*-tests. All the bar graphs represent the means (±SD) of triplicates of at least three independent experiments.

To understand the mechanism of action of I-CRP in lung cancer cells we further continue to characterize the type of cell death induced, through the analysis of important features associated with regulated cell death (RCD). DNA degradation is a common hallmark conserved in many types of RCD (Galluzzi et al., 2018), thus we assessed sub-G1 population in NSCLC cells after treatment with I-CRP. Results show DNA degradation, which was visible since 24 h of treatment with I-CRP, reaching up to 60% of DNA degradation in A549 cells after 48 h of treatment (Fig. 4A) and up to 80% in A427 cells (Fig. 4B) after 72 h of treatment.

Mitochondrial damage is another feature associated with RCD (Kroemer & Reed, 2000; Galluzzi et al., 2018), therefore we assessed mitochondrial membrane potential (Δψm) after treatment with I-CRP, using TMRE staining. We found that treatment with I-CRP leads to the loss of Δψm in A549 (Fig. 4C) and A427 (Fig. 4D) cells. Remarkably, the CC_50_ resulted in a Δψm loss that was near 70% in both cases (A549: 73%; A427: 67%).

Both features, DNA degradation and mitochondrial damage, are common characteristics of apoptotic cell death (Galluzzi et al., 2018), however, we have recently demonstrated that I-CRP induces caspase-independent RCD in HeLa cells (Martínez-Torres et al., 2018a). Therefore, to evaluate if this also occurs in NSCLC cell lines, we pre-incubated A549 and A427 cells with a pan caspase-inhibitor (QVD) before treating cells with I-CRP or etoposide, an apoptosis inductor (Montecucco, Zanetta & Biamonti, 2015) used as a positive control, and analyzed cell death. Results show that, contrary to the etoposide, in which cell death is significantly inhibited by QVD, I-CRP-induced cell death is not prevented by this pan-caspase inhibitor in A549 (Fig. 4E) nor A427 cells (Fig. 4F). These results show that even though I-CRP leads to DNA degradation and mitochondrial damage, caspase-independence is a shared feature of I-CRP-induced cell death in NSCLC cells as well.

### I-CRP induces ROS-dependent cell death

**Figure 5:**
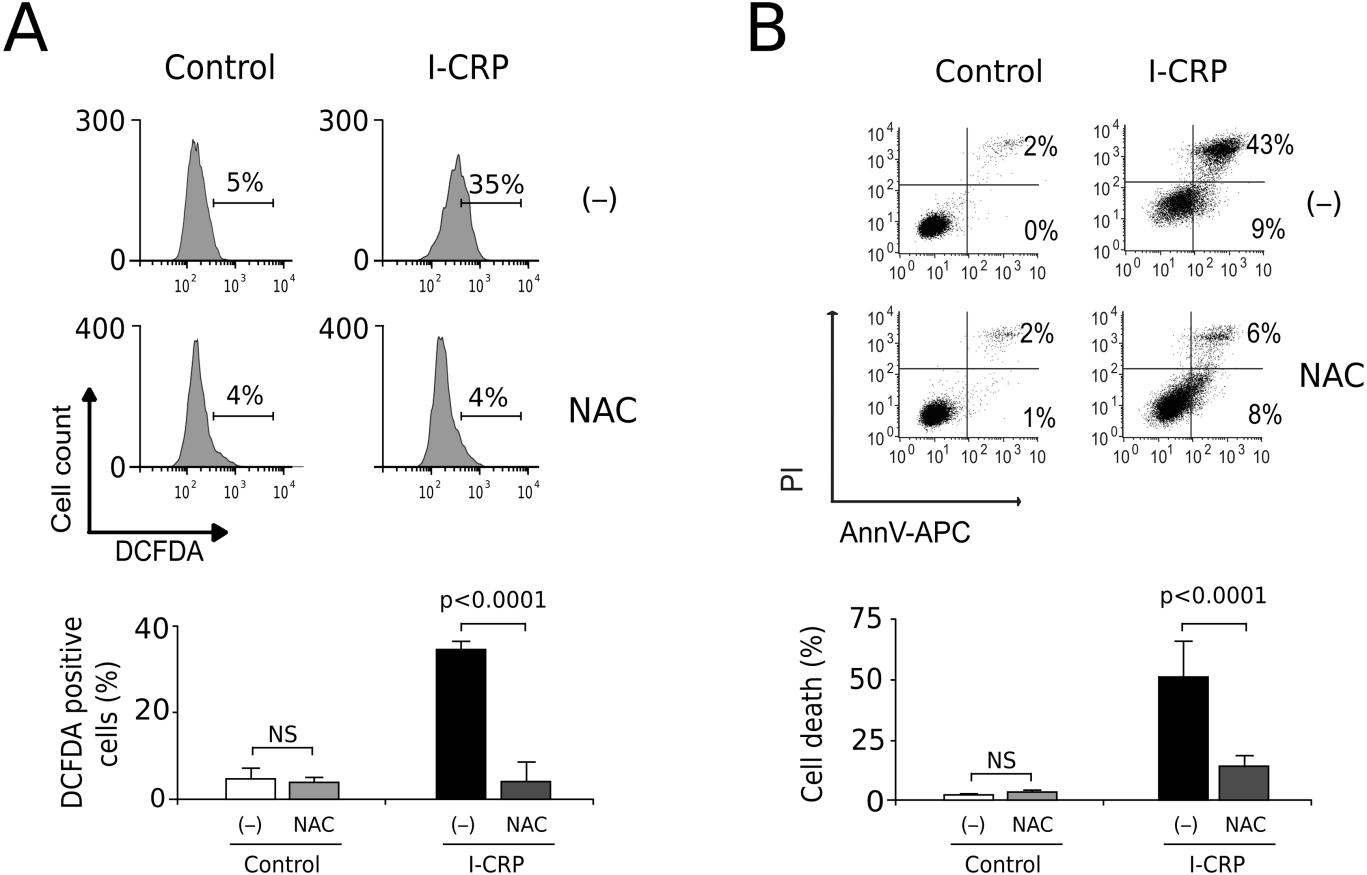
I-CRP induces ROS production and ROS-dependent cell death in A549 cells. (A) ROS production was measured by flow cytometry through DCFDA staining of A549 cells left alone (−) or pre-treated with NAC (5 mM) for 4 h, and then left untreated (Control) or treated with I-CRP (1.5 U/mL) for 24 h. Percentages in the representative histograms indicate the percentage of cells with high DCFDA fluorescence intensity. (B) Cell death was measured by flow cytometry through the Ann-V-APC/PI staining of A549 cells treated in the same conditions as in A. Percentages indicate the number of cells with Ann-V-APC^+^/PI^+^ and Ann-V-APC^+^/PI^−^ staining. The bar graphs in A and B represent the means (±SD) of triplicates of at least three independent experiments. Statistical analyses were performed using *t*-tests.

Since caspases were not responsible of cell death induced by I-CRP, and we observed damage of the mitochondria, a major source of ROS in the cell, we decided to evaluate whether I-CRP-induced cell death in lung cancer cells occurred due to ROS production. First, we assessed ROS production through DCFDA staining. In Fig. 5A we can observe that I-CRP induces ROS production, which can be significantly inhibited by the antioxidant NAC. Furthermore, ROS inhibition by NAC leads to a significant diminution of I-CRP-induced cell death (Fig. 5B), indicating that I-CRP induces a type of cell death that is dependent on ROS production.

## Discussion

Dialyzable leukocyte extracts (DLE) have been principally studied for their immunomodulatory properties, especially in infections and auto-immune diseases (Arnaudov & Kostova, 2015). However, different types of DLE have also demonstrated to have anti-tumor activity in pre-clinical studies. For example, swine DLE has shown to synergize with carmustine to reduce tumor volume in rats with heterotopic glioma tumors (Pineda et al., 2005). Similarly, I-CRP has proved anticancer effects when used alone or combined with chemotherapies in mice. In murine melanoma, I-CRP has shown to have *in vivo* anti-angiogenic activity (Franco-Molina et al., 2010) and to induce immunogenic cell death (Rodríguez-Salazar et al., 2017). Moreover, different DLE were used as adjuvants combined with conventional therapy in cancer clinical studies many years ago (Fudenberg, 1976; Pilotti et al., 1996; Pizza et al., 1996). In non-small cell lung cancer (NSCLC) patients undergoing classical radiotherapy/chemotherapy regimen, showed that I-CRP administration increased leukocyte counts and improved patients’ quality of life (Franco-Molina et al., 2008). However, even though it has also shown to be able to reduce cell viability in several cancer cell lines (Franco-Molina et al., 2006; Franco-Molina et al., 2010; Sierra-Rivera et al., 2016; Martínez-Torres et al., 2018a), when used alone, its mechanism of cytotoxicity in NSCLC cells had not been previously assessed until this study.

Here, we showed that I-CRP induced similar cytotoxicity in different NSCLC cell lines, independently of the diverse genetic aberrations of the cell lines tested (Lu & Arthur, 1992; Berger et al., 1999; Matsumoto et al., 2007; Luque et al., 2012; American Type Culture Collection, 2014; Barrera-Rodríguez & Fuentes, 2015). Additionally, although A549 and A427 had previously shown different susceptibility to several chemotherapies and other anti-cancer agents (Lu & Arthur, 1992; Odonkor & Achilefu, 2009; Luque et al., 2012; Garon et al., 2014), here we found that they shared similar features of cell death when treated with I-CRP. The conserved mechanism of cytotoxicity induced by I-CRP in A549 and A427 cells includes cell cycle arrest and cell death through mitochondrial and DNA damage. These features seem to be preserved also in other types of cancer cell lines, as we recently demonstrated that it also induced cell cycle arrest and cell death with DNA and mitochondrial alterations in HeLa cells (Martínez-Torres et al., 2018a).

The first biochemical and molecular analyses on the mechanism of cell death induced by I-CRP suggested that it induced apoptosis, since DNA degradation was observed in MCF-7 cells together with the suppression of *p53*, *bag-1*, *c-myc*, *bax*, *bcl-2*, and *bad* mRNA expression (Franco-Molina et al., 2006; Mendoza-Gamboa et al., 2008). However, in the present study we show that, although I-CRP also induces DNA-degradation in A549 and A427 cells, cell death is independent of caspases, as shown by our results using the pan-caspase inhibitor Q-VD-OPH (Caserta et al., 2003). This indicates that I-CRP induces a caspase-independent type of cell death, which is consistent with our previous results in HeLa cells, where we also found caspases to be dispensable for cell death induced by I-CRP (Martínez-Torres et al., 2018a). The Inhibition of the enzymatic activity of caspases is a widely used experimental approach to study the caspase dependence of cell death (Kögel & Prehn, 2013), as caspase activation can occur in other cellular processes that are not necessarily related to cell death (Nhan, Liles & Schwartz, 2006; Lamkanfi et al., 2007). Thus, even though I-CRP-induced cell death shares some biochemical features with apoptosis (Galluzzi et al., 2018), such as DNA degradation and mitochondrial damage (Martínez-Torres et al., 2018a), it induces caspase-independent cell death in NSCLC cell lines, as well as in HeLa cells.

In the present study we found that ROS production is critical for cell death induction by I-CRP in NSCLC cancer cells. Recently, we reported a similar result in HeLa cells, uncovering the importance of ROS production in I-CRP-induced cell death. Several cell death mechanisms have been described to be caspase-independent but ROS production-dependent processes. For example, a regulated cell death modality characterized by being a caspase-independent and ROS-dependent process was recently described under the name of oxeiptosis (Holze et al., 2018). Cytotoxicity induced by I-CRP has some similarities with this type of cell death, as it induces DNA degradation, mitochondrial depolarization and ROS production (Holze et al., 2018; Martínez-Torres et al., 2018a). However, oxeiptosis cell death signaling involves interactions among KEAP1, the phosphatase PGAM5, and the pro-apoptotic factor AIFM1. Thus, further research evaluating these molecules should be done in order to determine whether the cell death mechanism induced by I-CRP is oxeiptosis, especially because ROS production is also linked to other types of caspase-independent cell death modalities (Galluzzi et al., 2018). One of the best known is parthanatos, which can be induced by ROS overproduction, causing oxygen-glucose deprivation accompanied by PARP-1 upregulation, accumulation of PAR polymers, decline of mitochondrial membrane potential, and nuclear translocation of AIFM1 leading to DNA degradation (Fatokun, Dawson & Dawson, 2014; Wang et al., 2018). ROS inhibition with the antioxidant NAC showed to inhibit all of these effects (Fatokun, Dawson & Dawson, 2014; Wang et al., 2018). Thus, more detailed analysis should be done to better understand the role of ROS and the molecular mechanism of cell death induced by I-CRP.

## Conclusions

Overall, this work shows that I-CRP is cytotoxic in different lung cancer cell lines in a similar manner. We found that its cytotoxic activity occurs through cell death induction and cell cycle arrest. I-CRP-induced cell death is accompanied by DNA degradation, mitochondrial damage and ROS production. Furthermore, the use of the antioxidant NAC prevents I-CRP-induced ROS production and cell death, indicating that I-CRP induces ROS-dependent cell death in lung cancer cells. This work opens the way to further analyze the compatibility of this atypical type of cell death with first line therapies against lung cancer.

##  Supplemental Information

10.7717/peerj.7759/supp-1 Data S1 Raw data: Viability obtained by MTT assay

10.7717/peerj.7759/supp-2 Data S2 Raw data: Cell death (%) by Ann/PI staining

10.7717/peerj.7759/supp-3 Data S3 Raw data: Cells in cell cycle (%) in A549 and A427 cells

10.7717/peerj.7759/supp-4 Data S4 Raw data: Cells (%) in sub-G1 phase

10.7717/peerj.7759/supp-5 Figure S1 Raw data: I-CRP-induced ROS productionand ROS-dependent cell death

## Additional Information and Declarations

### Data Availability

The following information was supplied regarding data availability: The raw data is available as Supplemental Files.
