# Supplementary material for: Cytotoxic activity of IMMUNEPOTENT CRP against non-small cell lung cancer cell lines
Source: PeerJ. 2019 Sep 27;7:e7759. doi: 10.7717/peerj.7759 (PMC6768219; doi:10.7717/peerj.7759)
Supplement: Data S1 [file peerj-07-7759-s001.docx]

**Sup. 1 A. A549 cell viability evaluation by MTT.**

| **24 h** | | | | | | | | | |
| --- | --- | --- | --- | --- | --- | --- | --- | --- | --- |
|  |  |  |  |  |  |  |  |  |  |
| **Exp. 1** | **0** | **0.25** | **0.5** | **0.75** | **1** | **1.25** | **1.5** | **1.75** | **2** |
|  | 0.618 | 0.498 | 0.441 | 0.431 | 0.376 | 0.322 | 0.283 | 0.207 | 0.206 |
|  | 0.663 | 0.497 | 0.454 | 0.411 | 0.409 | 0.347 | 0.288 | 0.181 | 0.184 |
|  | 0.637 | 0.503 | 0.465 | 0.485 | 0.468 | 0.318 | 0.294 | 0.178 | 0.161 |
| x abs | 0.639 | 0.499 | 0.453 | 0.442 | 0.418 | 0.329 | 0.289 | 0.189 | 0.184 |
| Viability | 100% | 78% | 71% | 69% | 65% | 51% | 45% | 30% | 29% |
| Std. Dev. | 2% | 0% | 1% | 4% | 5% | 2% | 1% | 2% | 2% |
|  |  |  |  |  |  |  |  |  |  |
| **Exp. 2** | **0** | **0.25** | **0.5** | **0.75** | **1** | **1.25** | **1.5** | **1.75** | **2** |
|  | 0.418 | 0.305 | 0.277 | 0.332 | 0.229 | 0.200 | 0.138 | 0.138 | 0.126 |
|  | 0.448 | 0.356 | 0.276 | 0.265 | 0.283 | 0.233 | 0.126 | 0.154 | 0.117 |
|  | 0.365 | 0.321 | 0.300 | 0.315 | 0.299 | 0.213 | 0.124 | 0.124 | 0.122 |
| x abs | 0.410 | 0.327 | 0.284 | 0.304 | 0.270 | 0.215 | 0.129 | 0.139 | 0.122 |
| Viability | 100% | 80% | 69% | 74% | 66% | 52% | 32% | 34% | 30% |
| Std. Dev. | 4% | 3% | 1% | 3% | 4% | 2% | 1% | 2% | 1% |
|  |  |  |  |  |  |  |  |  |  |
| **Exp. 3** | **0** | **0.25** | **0.5** | **0.75** | **1** | **1.25** | **1.5** | **1.75** | **2** |
|  | 0.512 | 0.463 | 0.390 | 0.318 | 0.377 | 0.358 | 0.161 | 0.141 | 0.158 |
|  | 0.569 | 0.394 | 0.390 | 0.295 | 0.403 | 0.238 | 0.135 | 0.128 | 0.158 |
|  | 0.425 | 0.367 | 0.387 | 0.289 | 0.337 | 0.322 | 0.142 | 0.13 | 0.121 |
| x abs | 0.502 | 0.408 | 0.389 | 0.301 | 0.372 | 0.306 | 0.146 | 0.133 | 0.146 |
| Viability | 100% | 81% | 77% | 60% | 74% | 61% | 29% | 26% | 29% |
| Std. Dev. | 7% | 5% | 0% | 2% | 3% | 6% | 1% | 1% | 2% |
|  |  |  |  |  |  |  |  |  |  |
|  |  |  |  |  |  |  |  |  |  |
| Mean | 0 | 0.25 | 0.5 | 0.75 | 1 | 1.25 | 1.5 | 1.75 | 2 |
| Viability | **100%** | **80%** | **73%** | **68%** | **68%** | **55%** | **35%** | **30%** | **29%** |
| Std. Dev. | 9% | 6% | 2% | 5% | 7% | 7% | 2% | 3% | 3% |

| **48 h** | | | | | | | | | |
| --- | --- | --- | --- | --- | --- | --- | --- | --- | --- |
|  |  |  |  |  |  |  |  |  |  |
| **Exp.1** | **0** | **0.25** | **0.5** | **0.75** | **1** | **1.25** | **1.5** | **1.75** | **2** |
|  | 0.977 | 0.733 | 0.559 | 0.378 | 0.288 | 0.289 | 0.13 | 0.165 | 0.137 |
|  | 1.11 | 0.7 | 0.638 | 0.399 | 0.28 | 0.319 | 0.275 | 0.183 | 0.161 |
|  | 1.122 | 0.8 | 0.708 | 0.528 | 0.403 | 0.27 | 0.271 | 0.239 | 0.158 |
| x abs | 1.070 | 0.744 | 0.635 | 0.435 | 0.324 | 0.293 | 0.225 | 0.196 | 0.152 |
| Viability | 100% | 70% | 59% | 41% | 30% | 27% | 21% | 18% | 14% |
| Std. Dev. | 8% | 5% | 7% | 8% | 7% | 2% | 8% | 4% | 1% |
|  |  |  |  |  |  |  |  |  |  |
| **Exp. 2** | **0** | **0.25** | **0.5** | **0.75** | **1** | **1.25** | **1.5** | **1.75** | **2** |
|  | 1.093 | 0.778 | 0.649 | 0.518 | 0.319 | 0.278 | 0.183 | 0.099 | 0.088 |
|  | 1.083 | 0.711 | 0.668 | 0.567 | 0.339 | 0.305 | 0.221 | 0.268 | 0.128 |
|  | 0.999 | 0.709 | 0.607 | 0.534 | 0.346 | 0.281 | 0.345 | 0.222 | 0.239 |
| x abs | 1.058 | 0.733 | 0.641 | 0.540 | 0.335 | 0.288 | 0.250 | 0.196 | 0.152 |
| Viability | 100% | 69% | 61% | 51% | 32% | 27% | 24% | 19% | 14% |
| Std. Dev. | 5% | 4% | 3% | 2% | 1% | 1% | 8% | 9% | 8% |
|  |  |  |  |  |  |  |  |  |  |
| **Exp. 3** | **0** | **0.25** | **0.5** | **0.75** | **1** | **1.25** | **1.5** | **1.75** | **2** |
|  | 0.828 | 0.667 | 0.558 | 0.502 | 0.294 | 0.292 | 0.26 | 0.155 | 0.104 |
|  | 0.877 | 0.613 | 0.525 | 0.504 | 0.248 | 0.281 | 0.211 | 0.161 | 0.172 |
|  | 0.84 | 0.584 | 0.484 | 0.468 | 0.248 | 0.234 | 0.217 | 0.221 | 0.124 |
| x abs | 0.848 | 0.621 | 0.522 | 0.491 | 0.263 | 0.269 | 0.229 | 0.179 | 0.133 |
| Viability | 100% | 73% | 62% | 58% | 31% | 32% | 27% | 21% | 16% |
| Std. Dev. | 3% | 4% | 4% | 2% | 3% | 3% | 3% | 4% | 3% |
|  |  |  |  |  |  |  |  |  |  |
|  |  |  |  |  |  |  |  |  |  |
| Mean | 0 | 0.25 | 0.5 | 0.75 | 1 | 1.25 | 1.5 | 1.75 | 2 |
| Viability | **100%** | **71%** | **61%** | **50%** | **31%** | **29%** | **24%** | **19%** | **15%** |
| Std. Dev. | 10% | 8% | 9% | 9% | 8% | 4% | 12% | 10% | 9% |

| **72 h** | | | | | | | | | |
| --- | --- | --- | --- | --- | --- | --- | --- | --- | --- |
|  |  |  |  |  |  |  |  |  |  |
| **Exp. 1** | **0** | **0.25** | **0.5** | **0.75** | **1** | **1.25** | **1.5** | **1.75** | **2** |
|  | 0.882 | 0.622 | 0.494 | 0.453 | 0.245 | 0.163 | 0.098 | 0.075 | 0.081 |
|  | 0.742 | 0.608 | 0.522 | 0.337 | 0.267 | 0.144 | 0.089 | 0.074 | 0.078 |
|  | 0.812 | 0.63 | 0.549 | 0.387 | 0.297 | 0.178 | 0.109 | 0.074 | 0.074 |
| x abs | 0.812 | 0.620 | 0.522 | 0.392 | 0.270 | 0.162 | 0.099 | 0.074 | 0.078 |
| Viability | 100% | 76% | 64% | 48% | 33% | 20% | 12% | 9% | 10% |
| Std. Dev. | 7% | 1% | 3% | 6% | 3% | 2% | 1% | 0% | 0% |
|  |  |  |  |  |  |  |  |  |  |
| **Exp. 2** | **0** | **0.25** | **0.5** | **0.75** | **1** | **1.25** | **1.5** | **1.75** | **2** |
|  | 0.746 | 0.549 | 0.395 | 0.308 | 0.221 | 0.087 | 0.078 | 0.059 | 0.052 |
|  | 0.732 | 0.565 | 0.417 | 0.267 | 0.227 | 0.083 | 0.081 | 0.056 | 0.052 |
|  | 0.748 | 0.524 | 0.408 | 0.289 | 0.202 | 0.08 | 0.087 | 0.059 | 0.058 |
| x abs | 0.742 | 0.546 | 0.407 | 0.288 | 0.217 | 0.083 | 0.082 | 0.058 | 0.054 |
| Viability | 100% | 74% | 55% | 39% | 29% | 11% | 11% | 8% | 7% |
| Std. Dev. | 1% | 2% | 1% | 2% | 1% | 0% | 0% | 0% | 0% |
|  |  |  |  |  |  |  |  |  |  |
| **Exp. 3** | **0** | **0.25** | **0.5** | **0.75** | **1** | **1.25** | **1.5** | **1.75** | **2** |
|  | 0.889 | 0.595 | 0.451 | 0.33 | 0.257 | 0.136 | 0.074 | 0.053 | 0.059 |
|  | 0.896 | 0.644 | 0.425 | 0.341 | 0.266 | 0.109 | 0.078 | 0.053 | 0.056 |
|  | 0.879 | 0.618 | 0.49 | 0.351 | 0.272 | 0.116 | 0.08 | 0.057 | 0.067 |
| x abs | 0.888 | 0.619 | 0.455 | 0.341 | 0.265 | 0.120 | 0.077 | 0.054 | 0.061 |
| Viability | 100% | 70% | 51% | 38% | 30% | 14% | 9% | 6% | 7% |
| Std. Dev. | 1% | 2% | 3% | 1% | 1% | 1% | 0% | 0% | 1% |
|  |  |  |  |  |  |  |  |  |  |
|  |  |  |  |  |  |  |  |  |  |
| Mean | 0 | 0.25 | 0.5 | 0.75 | 1 | 1.25 | 1.5 | 1.75 | 2 |
| Viability | **100%** | **73%** | **57%** | **42%** | **31%** | **15%** | **11%** | **8%** | **8%** |
| Std. Dev. | 7% | 3% | 4% | 6% | 3% | 2% | 1% | 0% | 1% |

**Sup. 1 B. A427 cell viability evaluation by MTT.**

| 24 h | | | | | | | | | |
| --- | --- | --- | --- | --- | --- | --- | --- | --- | --- |
|  |  |  |  |  |  |  |  |  |  |
| **Exp. 1** | 0 | 0.25 | 0.5 | 0.75 | 1 | 1.25 | 1.5 | 1.75 | 2 |
|  | 0.851 | 0.783 | 0.718 | 0.687 | 0.541 | 0.222 | 0.179 | 0.178 | 0.185 |
|  | 0.700 | 0.770 | 0.715 | 0.751 | 0.595 | 0.187 | 0.198 | 0.188 | 0.186 |
|  |  | 0.719 | 0.771 | 0.640 | 0.578 | 0.253 | 0.179 | 0.181 | - |
| x abs | 0.776 | 0.757 | 0.735 | 0.693 | 0.571 | 0.221 | 0.185 | 0.182 | 0.186 |
| Viability | 100% | 98% | 95% | 89% | 74% | 28% | 24% | 24% | 24% |
| Std. Dev. | 11% | 3% | 3% | 6% | 3% | 3% | 1% | 1% | 11% |
|  |  |  |  |  |  |  |  |  |  |
| **Exp. 2** | 0 | 0.25 | 0.5 | 0.75 | 1 | 1.25 | 1.5 | 1.75 | 2 |
|  | 0.310 | 0.202 | 0.283 | 0.163 | 0.262 | 0.215 | 0.157 | 0.138 | 0.150 |
|  | 0.245 | 0.223 | 0.234 | 0.240 | 0.201 | 0.180 | 0.139 | 0.161 | - |
|  | 0.270 | 0.251 | 0.179 | 0.265 | 0.195 | 0.180 | 0.138 | 0.137 | 0.148 |
| x abs | 0.275 | 0.225 | 0.232 | 0.223 | 0.219 | 0.192 | 0.145 | 0.145 | 0.149 |
| Viability | 100% | 82% | 84% | 81% | 80% | 70% | 53% | 53% | 54% |
| Std. Dev. | 3% | 2% | 5% | 5% | 4% | 2% | 1% | 1% | 9% |
|  |  |  |  |  |  |  |  |  |  |
| **Exp. 3** | 0 | 0.25 | 0.5 | 0.75 | 1 | 1.25 | 1.5 | 1.75 | 2 |
|  | 0.655 | 0.487 | 0.430 | 0.422 | 0.355 | 0.240 | 0.126 | 0.134 | 0.144 |
|  | 0.569 | 0.433 | 0.428 | 0.379 | 0.376 | 0.208 | 0.159 | 0.127 | 0.121 |
|  | 0.586 | 0.430 | 0.385 | 0.313 | 0.329 | 0.245 | 0.120 | 0.125 | 0.124 |
| x abs | 0.603 | 0.450 | 0.414 | 0.371 | 0.353 | 0.231 | 0.135 | 0.129 | 0.130 |
| Viability | 100% | 75% | 69% | 62% | 59% | 38% | 22% | 21% | 21% |
| Std. Dev. | 5% | 3% | 3% | 5% | 2% | 2% | 2% | 0% | 1% |
|  |  |  |  |  |  |  |  |  |  |
|  |  |  |  |  |  |  |  |  |  |
| Mean | 0 | 0.25 | 0.5 | 0.75 | 1 | 1.25 | 1.5 | 1.75 | 2 |
| Viability | **100%** | **85%** | **83%** | **77%** | **71%** | **45%** | **33%** | **33%** | **33%** |
| Std. Dev. | 12% | 5% | 7% | 9% | 5% | 4% | 3% | 2% | 14% |

| 48 h | | | | | | | | | |
| --- | --- | --- | --- | --- | --- | --- | --- | --- | --- |
|  |  |  |  |  |  |  |  |  |  |
| **Exp. 1** | **0** | **0.25** | **0.5** | **0.75** | **1** | **1.25** | **1.5** | **1.75** | **2** |
|  | 0.364 | 0.258 | 0.296 | 0.249 | 0.198 | 0.160 | 0.158 | 0.110 | 0.088 |
|  | 0.382 | 0.326 | 0.237 | 0.222 | 0.225 | 0.169 | 0.161 | 0.114 | 0.104 |
|  | 0.487 | 0.392 | 0.241 | 0.311 | 0.219 | 0.161 | 0.154 | 0.121 | 0.068 |
| x abs | 0.411 | 0.325 | 0.258 | 0.261 | 0.214 | 0.163 | 0.158 | 0.115 | 0.087 |
| Viability | 100% | 79% | 63% | 63% | 52% | 40% | 38% | 28% | 21% |
| Std. Dev. | 7% | 7% | 3% | 5% | 1% | 0% | 0% | 1% | 2% |
|  |  |  |  |  |  |  |  |  |  |
| **Exp. 2** | **0** | **0.25** | **0.5** | **0.75** | **1** | **1.25** | **1.5** | **1.75** | **2** |
|  | 0.625 | 0.538 | 0.408 | 0.351 | 0.368 | 0.255 | 0.167 | 0.097 | 0.094 |
|  | 0.626 | 0.423 | 0.406 | 0.360 | 0.324 | 0.226 | 0.107 | 0.098 | 0.086 |
|  | 0.615 | 0.454 | 0.348 | 0.284 | 0.262 | 0.197 | 0.157 | 0.085 | 0.086 |
| x abs | 0.622 | 0.472 | 0.387 | 0.332 | 0.318 | 0.226 | 0.144 | 0.093 | 0.089 |
| Viability | 100% | 76% | 62% | 53% | 51% | 36% | 23% | 15% | 14% |
| Std. Dev. | 1% | 6% | 3% | 4% | 5% | 3% | 3% | 1% | 0% |
|  |  |  |  |  |  |  |  |  |  |
| **Exp. 3** | **0** | **0.25** | **0.5** | **0.75** | **1** | **1.25** | **1.5** | **1.75** | **2** |
|  | 0.828 | 0.677 | 0.578 | 0.542 | 0.519 | 0.395 | 0.285 | 0.160 | 0.144 |
|  | 0.877 | 0.623 | 0.545 | 0.544 | 0.473 | 0.384 | 0.236 | 0.166 | 0.212 |
|  | 0.840 | 0.594 | 0.504 | 0.508 | 0.473 | 0.337 | 0.247 | 0.226 | 0.164 |
| x abs | 0.848 | 0.631 | 0.542 | 0.531 | 0.488 | 0.372 | 0.256 | 0.184 | 0.173 |
| Viability | 100% | 74% | 64% | 63% | 58% | 44% | 30% | 22% | 20% |
| Std. Dev. | 3% | 4% | 4% | 2% | 3% | 3% | 3% | 4% | 3% |
|  |  |  |  |  |  |  |  |  |  |
|  |  |  |  |  |  |  |  |  |  |
| Mean | 0 | 0.25 | 0.5 | 0.75 | 1 | 1.25 | 1.5 | 1.75 | 2 |
| Viability | **100%** | **76%** | **63%** | **60%** | **54%** | **40%** | **31%** | **22%** | **19%** |
| Std. Dev. | 7% | 10% | 6% | 6% | 6% | 4% | 4% | 4% | 4% |

| 72 h | | | | | | | | | |
| --- | --- | --- | --- | --- | --- | --- | --- | --- | --- |
|  |  |  |  |  |  |  |  |  |  |
| **Exp. 1** | **0** | **0.25** | **0.5** | **0.75** | **1** | **1.25** | **1.5** | **1.75** | **2** |
|  | 0.727 | 0.505 | 0.428 | 0.328 | 0.248 | 0.103 | 0.085 | 0.098 | 0.086 |
|  | 0.647 | 0.498 | 0.425 | 0.362 | 0.272 | 0.091 | 0.084 | 0.087 | 0.089 |
|  | 0.760 | 0.500 | 0.466 | 0.375 | 0.220 | 0.096 | 0.090 | 0.089 | 0.088 |
| x abs | 0.711 | 0.501 | 0.440 | 0.355 | 0.247 | 0.097 | 0.086 | 0.091 | 0.088 |
| Viability | 100% | 70% | 62% | 50% | 35% | 14% | 12% | 13% | 12% |
| Std. Dev. | 6% | 0% | 2% | 2% | 3% | 1% | 0% | 1% | 0% |
|  |  |  |  |  |  |  |  |  |  |
| **Exp. 2** | **0** | **0.25** | **0.5** | **0.75** | **1** | **1.25** | **1.5** | **1.75** | **2** |
|  | 0.513 | 0.239 | 0.200 | 0.217 | 0.189 | 0.128 | 0.047 | 0.047 | 0.031 |
|  | 0.524 | 0.329 | 0.255 | 0.214 | 0.176 | 0.136 | 0.059 | 0.038 | 0.027 |
|  | 0.469 | 0.306 | 0.251 | 0.207 | 0.168 | 0.135 | 0.063 | 0.047 | 0.033 |
| x abs | 0.502 | 0.291 | 0.235 | 0.212 | 0.177 | 0.133 | 0.056 | 0.044 | 0.030 |
| Viability | 100% | 58% | 47% | 42% | 35% | 26% | 11% | 9% | 6% |
| Std. Dev. | 3% | 5% | 3% | 1% | 1% | 0% | 1% | 1% | 0% |
|  |  |  |  |  |  |  |  |  |  |
| **Exp. 3** | **0** | **0.25** | **0.5** | **0.75** | **1** | **1.25** | **1.5** | **1.75** | **2** |
|  | 0.953 | 0.612 | 0.551 | 0.48 | 0.346 | 0.191 | 0.103 | 0.105 | 0.077 |
|  | 0.92 | 0.637 | 0.535 | 0.477 | 0.334 | 0.182 | 0.118 | 0.094 | 0.08 |
|  | 0.987 | 0.668 | 0.543 | 0.474 | 0.324 | 0.197 | 0.114 | 0.118 | 0.074 |
| x abs | 0.953 | 0.639 | 0.543 | 0.477 | 0.335 | 0.190 | 0.112 | 0.106 | 0.077 |
| Viability | 100% | 67% | 57% | 50% | 35% | 20% | 12% | 11% | 8% |
| Std. Dev. | 3% | 3% | 1% | 0% | 1% | 1% | 1% | 1% | 0% |
|  |  |  |  |  |  |  |  |  |  |
|  |  |  |  |  |  |  |  |  |  |
| Mean | 0 | 0.25 | 0.5 | 0.75 | 1 | 1.25 | 1.5 | 1.75 | 2 |
| Viability | **100%** | **69%** | **59%** | **50%** | **35%** | **17%** | **12%** | **12%** | **10%** |
| Std. Dev. | 7% | 5% | 4% | 2% | 3% | 1% | 1% | 1% | 0% |

**Sup. 1 C. CALU-1 cell viability evaluation by MTT.**

| **24 h** | | | | | | | | | |
| --- | --- | --- | --- | --- | --- | --- | --- | --- | --- |
|  |  |  |  |  |  |  |  |  |  |
| Exp. 1 | 0 | 0.25 | 0.5 | 0.75 | 1 | 1.25 | 1.5 | 1.75 | 2 |
|  | 0.618 | 0.55 | 0.474 | 0.475 | 0.376 | 0.251 | 0.244 | 0.207 | - |
|  | 0.663 | 0.53 | 0.522 | 0.473 | 0.389 | 0.222 | 0.181 | 0.181 | 0.181 |
|  | 0.637 | 0.538 | 0.503 | 0.471 | 0.359 | 0.257 | 0.18 | 0.178 | 0.165 |
| x̃ abs | 0.639 | 0.539 | 0.500 | 0.473 | 0.375 | 0.243 | 0.202 | 0.189 | 0.173 |
| Viability | 100% | 84% | 78% | 74% | 59% | 38% | 32% | 30% | 27% |
| Std. Dev. | 2% | 1% | 2% | 0% | 2% | 2% | 4% | 2% | 10% |
|  |  |  |  |  |  |  |  |  |  |
| Exp. 2 | 0 | 0.25 | 0.5 | 0.75 | 1 | 1.25 | 1.5 | 1.75 | 2 |
|  | - | - | 0.756 | 0.649 | 0.661 | - | - | - | 0.206 |
|  | 0.884 | 0.626 | 0.696 | 0.615 | 0.612 | 0.588 | - | - | 0.283 |
|  | 0.843 | 0.610 | 0.649 | 0.539 | 0.612 | 0.549 | - | - | 0.249 |
| x̃ abs | 0.864 | 0.618 | 0.700 | 0.601 | 0.628 | 0.569 | - | - | 0.246 |
| Viability | 100% | 72% | 81% | 70% | 73% | 66% | - | - | 28% |
| Std. Dev | 50% | 36% | 5% | 6% | 3% | 33% | - | - | 4% |
|  |  |  |  |  |  |  |  |  |  |
| Exp. 3 | 0 | 0.25 | 0.5 | 0.75 | 1 | 1.25 | 1.5 | 1.75 | 2 |
|  | 0.578 | 0.486 | 0.476 | 0.488 | 0.4 | 0.368 | 0.155 | 0.158 | 0.158 |
|  | 0.578 | 0.504 | 0.528 | 0.469 | 0.411 | 0.367 | 0.167 | 0.158 | 0.181 |
|  | 0.682 | 0.495 | 0.476 | 0.472 | 0.464 | 0.401 | 0.165 | 0.162 | 0.189 |
| x̃ abs | 0.613 | 0.495 | 0.493 | 0.476 | 0.425 | 0.379 | 0.162 | 0.159 | 0.176 |
| Viability | 100% | 81% | 81% | 78% | 69% | 62% | 26% | 26% | 29% |
| Std. Dev. | 6% | 1% | 3% | 1% | 3% | 2% | 1% | 0% | 2% |
|  |  |  |  |  |  |  |  |  |  |
|  |  |  |  |  |  |  |  |  |  |
| Mean | 0 | 0.25 | 0.5 | 0.75 | 1 | 1.25 | 1.5 | 1.75 | 2 |
| Viability | **100%** | **79%** | **80%** | **74%** | **67%** | **55%** | **29%** | **28%** | **28%** |
| Std. Dev. | 50% | 36% | 7% | 6% | 5% | 33% | 4% | 2% | 11% |

| **48 h** | | | | | | | | | |
| --- | --- | --- | --- | --- | --- | --- | --- | --- | --- |
|  |  |  |  |  |  |  |  |  |  |
| Exp. 1 | 0 | 0.25 | 0.5 | 0.75 | 1 | 1.25 | 1.5 | 1.75 | 2 |
|  | 0.43 | 0.2585 | 0.2395 | 0.191 | 0.1565 | 0.1405 | - | - | - |
|  | 0.336 | 0.2025 | 0.199 | 0.171 | 0.151 | 0.141 | - | - | - |
|  | 0.3845 | 0.2575 | 0.1945 | 0.203 | 0.145 | 0.144 | - | - | - |
| x̃ abs | 0.384 | 0.240 | 0.211 | 0.188 | 0.151 | 0.142 | - | - | - |
| Viability | 100% | 62% | 55% | 49% | 39% | 37% | - | - | - |
| Std. Dev. | 5% | 3% | 2% | 2% | 1% | 0% | - | - | - |
|  |  |  |  |  |  |  |  |  |  |
| Exp. 2 | 0 | 0.25 | 0.5 | 0.75 | 1 | 1.25 | 1.5 | 1.75 | 2 |
|  | 0.885 | 0.723 | 0.549 | 0.399 | - | 0.299 | 0.256 | 0.21 | 0.162 |
|  | 0.898 | 0.74 | 0.469 | 0.386 | - | 0.241 | 0.267 | 0.183 | 0.145 |
|  | 0.839 | 0.642 | 0.56 | 0.340 | - | 0.255 | 0.238 | 0.179 | 0.146 |
| x̃ abs | 0.874 | 0.702 | 0.526 | 0.375 | - | 0.265 | 0.254 | 0.191 | 0.151 |
| Viability | 100% | 80% | 60% | 43% | - | 30% | 29% | 22% | 17% |
| Std. Dev. | 3% | 5% | 5% | 3% | - | 3% | 1% | 2% | 1% |
|  |  |  |  |  |  |  |  |  |  |
| Exp. 3 | 0 | 0.25 | 0.5 | 0.75 | 1 | 1.25 | 1.5 | 1.75 | 2 |
|  | 0.878 | 0.555 | 0.345 | 0.372 | 0.394 | 0.234 | 0.178 | 0.125 | 0.136 |
|  | 0.808 | 0.46 | 0.418 | 0.424 | 0.375 | 0.305 | 0.162 | 0.138 | 0.141 |
|  | 0.821 | 0.505 | 0.444 | 0.46 | 0.342 | 0.261 | 0.154 | 0.128 | 0.146 |
| x̃ abs | 0.836 | 0.507 | 0.402 | 0.419 | 0.370 | 0.267 | 0.165 | 0.130 | 0.141 |
| Viability | 100% | 61% | 48% | 50% | 44% | 32% | 20% | 16% | 17% |
| Std. Dev. | 4% | 5% | 5% | 4% | 3% | 4% | 1% | 1% | 0% |
|  |  |  |  |  |  |  |  |  |  |
|  |  |  |  |  |  |  |  |  |  |
| Exp. 4 | 0 | 0.25 | 0.5 | 0.75 | 1 | 1.25 | 1.5 | 1.75 | 2 |
|  | 0.864 | 0.617 | 0.576 | 0.516 | 0.436 | - | - | 0.247 | 0.029 |
|  | 0.878 | 0.596 | 0.584 | 0.502 | 0.489 | - | - | 0.23 | 0.03 |
|  | 0.846 | 0.721 | 0.567 | 0.547 | 0.509 | - | - | 0.252 | 0.042 |
| x̃ abs | 0.863 | 0.645 | 0.576 | 0.522 | 0.478 | - | - | 0.243 | 0.034 |
| Viability | 100% | 75% | 67% | 60% | 55% | - | - | 28% | 4% |
| Std. Dev. | 2% | 7% | 1% | 2% | 4% | - | - | 1% | 1% |
|  |  |  |  |  |  |  |  |  |  |
|  |  |  |  |  |  |  |  |  |  |
| Mean | 0 | 0.25 | 0.5 | 0.75 | 1 | 1.25 | 1.5 | 1.75 | 2 |
| Viability | **100%** | **70%** | **58%** | **51%** | **46%** | **33%** | **24%** | **22%** | **13%** |
| Std. Dev. | 7% | 10% | 8% | 6% | 5% | 5% | 2% | 2% | 1% |

| **72 h** | | | | | | | | | |
| --- | --- | --- | --- | --- | --- | --- | --- | --- | --- |
|  |  |  |  |  |  |  |  |  |  |
| **Exp. 1** | **0** | **0.25** | **0.5** | **0.75** | **1** | **1.25** | **1.5** | **1.75** | **2** |
|  | 0.576 | 0.470 | 0.357 | 0.249 | 0.160 | 0.046 | 0.034 | 0.042 | 0.041 |
|  | 0.542 | 0.365 | 0.376 | 0.296 | 0.189 | 0.046 | 0.044 | 0.041 | 0.044 |
|  | 0.595 | 0.420 | 0.424 | 0.273 | 0.181 | 0.047 | 0.040 | 0.041 | 0.041 |
| x abs | 0.571 | 0.418 | 0.386 | 0.273 | 0.177 | 0.046 | 0.039 | 0.041 | 0.042 |
| Viability | 100% | 73% | 68% | 48% | 31% | 8% | 7% | 7% | 7% |
| Std. Dev. | 3% | 5% | 3% | 2% | 1% | 0% | 1% | 0% | 0% |
|  |  |  |  |  |  |  |  |  |  |
| **Exp. 2** | **0** | **0.25** | **0.5** | **0.75** | **1** | **1.25** | **1.5** | **1.75** | **2** |
|  | 0.540 | 0.348 | - | 0.289 | 0.164 | 0.046 | 0.014 | 0.016 | 0.008 |
|  | 0.523 | 0.360 | 0.335 | 0.306 | 0.208 | 0.058 | 0.030 | 0.014 | 0.012 |
|  | 0.549 | 0.353 | 0.226 | 0.274 | 0.211 | 0.035 | 0.022 | 0.014 | 0.011 |
| x abs | 0.537 | 0.353 | 0.280 | 0.289 | 0.194 | 0.046 | 0.022 | 0.014 | 0.010 |
| Viability | 100% | 66% | 52% | 54% | 36% | 9% | 4% | 3% | 2% |
| Std. Dev. | 1% | 1% | 8% | 2% | 3% | 1% | 1% | 0% | 0% |
|  |  |  |  |  |  |  |  |  |  |
| **Exp. 3** | **0** | **0.25** | **0.5** | **0.75** | **1** | **1.25** | **1.5** | **1.75** | **2** |
|  | 0.667 | 0.577 | 0.428 | 0.337 | 0.253 | 0.031 | 0.027 | 0.039 | 0.044 |
|  | 0.662 | 0.487 | 0.378 | 0.347 | 0.260 | 0.026 | 0.031 | 0.027 | 0.037 |
|  | 0.650 | 0.501 | 0.421 | 0.424 | 0.232 | 0.028 | 0.028 | 0.043 | 0.035 |
| x abs | 0.659 | 0.521 | 0.409 | 0.369 | 0.248 | 0.028 | 0.028 | 0.036 | 0.038 |
| Viability | 100% | 79% | 62% | 56% | 38% | 4% | 4% | 5% | 6% |
| Std. Dev. | 1% | 5% | 3% | 5% | 1% | 0% | 0% | 1% | 0% |
|  |  |  |  |  |  |  |  |  |  |
|  |  |  |  |  |  |  |  |  |  |
| Mean | 0 | 0.25 | 0.5 | 0.75 | 1 | 1.25 | 1.5 | 1.75 | 2 |
| Viability | **100%** | **73%** | **61%** | **53%** | **35%** | **7%** | **5%** | **5%** | **5%** |
| Std. Dev. | 3% | 7% | 9% | 6% | 3% | 1% | 1% | 1% | 1% |

**Sup. 1 D. INER-51 cell viability evaluation by MTT.**

| **24 h** | | | | | | | | | |
| --- | --- | --- | --- | --- | --- | --- | --- | --- | --- |
|  |  |  |  |  |  |  |  |  |  |
| **Exp. 1** | **0** | **0.25** | **0.5** | **0.75** | **1** | **1.25** | **1.5** | **1.75** | **2** |
|  | 0.251 | 0.254 | 0.191 | 0.171 | 0.119 | 0.097 | 0.056 | 0.043 | 0.050 |
|  | 0.234 | 0.203 | 0.225 | 0.155 | 0.125 | 0.067 | 0.057 | 0.045 | 0.043 |
|  | 0.191 | 0.186 | 0.172 | 0.187 | 0.113 | 0.073 | 0.053 | 0.063 | 0.049 |
| x̃ abs | 0.225 | 0.214 | 0.196 | 0.171 | 0.119 | 0.079 | 0.055 | 0.050 | 0.047 |
| Viability | 100% | 95% | 87% | 76% | 53% | 35% | 25% | 22% | 21% |
| Std. Dev. | 3% | 4% | 3% | 2% | 1% | 2% | 0% | 1% | 0% |
|  |  |  |  |  |  |  |  |  |  |
| **Exp. 2** | **0** | **0.25** | **0.5** | **0.75** | **1** | **1.25** | **1.5** | **1.75** | **2** |
|  | 0.303 | 0.291 | 0.223 | 0.136 | 0.146 | 0.067 | 0.042 | 0.034 | 0.038 |
|  | 0.286 | 0.301 | 0.221 | 0.196 | 0.147 | 0.066 | 0.067 | 0.046 | 0.038 |
|  | 0.263 | 0.242 | 0.235 | 0.135 | 0.107 | 0.084 | 0.036 | 0.025 | 0.037 |
| x̃ abs | 0.284 | 0.278 | 0.226 | 0.156 | 0.133 | 0.072 | 0.048 | 0.035 | 0.038 |
| Viability | 100% | 98% | 80% | 55% | 47% | 25% | 17% | 12% | 13% |
| Std. Dev. | 2% | 3% | 1% | 3% | 2% | 1% | 2% | 1% | 0% |
|  |  |  |  |  |  |  |  |  |  |
| **Exp. 3** | **0** | **0.25** | **0.5** | **0.75** | **1** | **1.25** | **1.5** | **1.75** | **2** |
|  | 0.265 | 0.265 | 0.249 | 0.207 | 0.133 | 0.090 | 0.046 | 0.053 | 0.051 |
|  | 0.301 | 0.279 | 0.235 | 0.181 | 0.131 | 0.093 | 0.037 | 0.030 | 0.033 |
|  | 0.262 | 0.251 | 0.211 | 0.161 | 0.113 | 0.083 | 0.044 | 0.026 | 0.028 |
| x̃ abs | 0.276 | 0.265 | 0.232 | 0.183 | 0.126 | 0.089 | 0.042 | 0.036 | 0.037 |
| Viability | 100% | 96% | 84% | 66% | 46% | 32% | 15% | 13% | 14% |
| Std. Dev. | 2% | 1% | 2% | 2% | 1% | 1% | 0% | 1% | 1% |
|  |  |  |  |  |  |  |  |  |  |
|  |  |  |  |  |  |  |  |  |  |
| Mean | 0 | 0.25 | 0.5 | 0.75 | 1 | 1.25 | 1.5 | 1.75 | 2 |
| Viability | **100%** | **96%** | **84%** | **66%** | **48%** | **31%** | **19%** | **16%** | **16%** |
| Std. Dev. | 4% | 5% | 3% | 4% | 3% | 2% | 2% | 2% | 1% |

| **48 h** | | | | | | | | | |
| --- | --- | --- | --- | --- | --- | --- | --- | --- | --- |
|  |  |  |  |  |  |  |  |  |  |
| **Exp. 1** | **0** | **0.25** | **0.5** | **0.75** | **1** | **1.25** | **1.5** | **1.75** | **2** |
|  | 0.981 | 0.656 | 0.473 | 0.388 | 0.327 | 0.144 | 0.032 | 0.033 | 0.034 |
|  | 0.914 | 0.681 | 0.492 | 0.474 | 0.314 | 0.125 | 0.044 | 0.046 | 0.027 |
|  | 0.959 | 0.579 | 0.483 | 0.447 | 0.401 | 0.114 | 0.037 | 0.034 | 0.034 |
| x̃ abs | 0.951 | 0.639 | 0.483 | 0.436 | 0.347 | 0.128 | 0.038 | 0.038 | 0.032 |
| Viability | 100% | 67% | 51% | 46% | 37% | 13% | 4% | 4% | 3% |
| Std. Dev. | 3% | 5% | 1% | 4% | 5% | 2% | 1% | 1% | 0% |
|  |  |  |  |  |  |  |  |  |  |
| **Exp. 2** | **0** | **0.25** | **0.5** | **0.75** | **1** | **1.25** | **1.5** | **1.75** | **2** |
|  | 1.032 | 0.698 | 0.773 | 0.558 | 0.312 | 0.198 | 0.05 | 0.031 | 0.038 |
|  | 1.056 | 0.779 | 0.601 | 0.637 | 0.351 | 0.146 | 0.035 | 0.036 | 0.036 |
|  | 1.191 | 0.851 | 0.701 | 0.479 | 0.294 | 0.114 | 0.044 | 0.037 | 0.022 |
| x̃ abs | 1.093 | 0.776 | 0.692 | 0.558 | 0.319 | 0.153 | 0.043 | 0.035 | 0.032 |
| Viability | 100% | 71% | 63% | 51% | 29% | 14% | 4% | 3% | 3% |
| Std. Dev. | 9% | 8% | 9% | 8% | 3% | 4% | 1% | 0% | 1% |
|  |  |  |  |  |  |  |  |  |  |
| **Exp. 3** | **0** | **0.25** | **0.5** | **0.75** | **1** | **1.25** | **1.5** | **1.75** | **2** |
|  | 1.13 | 0.901 | 0.846 | 0.735 | 0.451 | 0.189 | 0.1 | 0.04 | 0.049 |
|  | 1.013 | 0.918 | 0.87 | 0.734 | 0.464 | 0.164 | 0.117 | 0.038 | 0.041 |
|  | 1.152 | 0.938 | 0.735 | 0.705 | 0.411 | 0.179 | 0.107 | 0.036 | 0.039 |
| x̃ abs | 1.098 | 0.919 | 0.817 | 0.725 | 0.442 | 0.177 | 0.108 | 0.038 | 0.043 |
| Viability | 100% | 84% | 74% | 66% | 40% | 16% | 10% | 3% | 4% |
| Std. Dev. | 7% | 2% | 7% | 2% | 3% | 1% | 1% | 0% | 1% |
|  |  |  |  |  |  |  |  |  |  |
|  |  |  |  |  |  |  |  |  |  |
| Mean | 0 | 0.25 | 0.5 | 0.75 | 1 | 1.25 | 1.5 | 1.75 | 2 |
| Viability | **100%** | **74%** | **63%** | **54%** | **35%** | **15%** | **6%** | **4%** | **3%** |
| Std. Dev. | 12% | 10% | 11% | 9% | 6% | 5% | 1% | 1% | 1% |

| **72 h** | | | | | | | | | |
| --- | --- | --- | --- | --- | --- | --- | --- | --- | --- |
|  |  |  |  |  |  |  |  |  |  |
| **Exp. 1** | **0** | **0.25** | **0.5** | **0.75** | **1** | **1.25** | **1.5** | **1.75** | **2** |
|  | 0.997 | 0.456 | 0.053 | 0.026 | 0.024 | 0.032 | 0.028 | 0.015 | 0.031 |
|  | 1.043 | 0.417 | 0.066 | 0.026 | 0.026 | 0.029 | 0.032 | 0.031 | 0.029 |
|  | 1.057 | 0.388 | 0.060 | 0.027 | 0.027 | 0.031 | 0.03 | 0.136 | 0.021 |
| x̃ abs | 1.032 | 0.420 | 0.060 | 0.026 | 0.026 | 0.031 | 0.030 | 0.061 | 0.027 |
| Viability | 100% | 41% | 6% | 3% | 2% | 3% | 3% | 6% | 3% |
| Std. Dev. | 3% | 3% | 1% | 0% | 0% | 0% | 0% | 7% | 1% |
|  |  |  |  |  |  |  |  |  |  |
| **Exp. 2** | **0** | **0.25** | **0.5** | **0.75** | **1** | **1.25** | **1.5** | **1.75** | **2** |
|  | 1.176 | 0.845 | 0.782 | 0.485 | 0.128 | 0.035 | 0.023 | 0.020 | 0.013 |
|  | 1.108 | 0.930 | 0.682 | 0.392 | 0.100 | 0.051 | 0.014 | 0.017 | 0.017 |
|  | 1.073 | 0.930 | 0.601 | 0.402 | 0.053 | 0.039 | 0.017 | 0.015 | 0.018 |
| x̃ abs | 1.119 | 0.902 | 0.688 | 0.426 | 0.094 | 0.042 | 0.018 | 0.017 | 0.016 |
| Viability | 100% | 81% | 62% | 38% | 8% | 4% | 2% | 2% | 1% |
| Std. Dev. | 5% | 5% | 9% | 5% | 4% | 1% | 0% | 0% | 0% |
|  |  |  |  |  |  |  |  |  |  |
|  |  |  |  |  |  |  |  |  |  |
| Mean | 0 | 0.25 | 0.5 | 0.75 | 1 | 1.25 | 1.5 | 1.75 | 2 |
| Viability | **100%** | **61%** | **34%** | **20%** | **5%** | **3%** | **2%** | **4%** | **2%** |
| Std. Dev. | 6% | 6% | 9% | 5% | 4% | 1% | 0% | 7% | 1% |
