# Supplementary material for: Cytotoxic activity of IMMUNEPOTENT CRP against non-small cell lung cancer cell lines
Source: PeerJ. 2019 Sep 27;7:e7759. doi: 10.7717/peerj.7759 (PMC6768219; doi:10.7717/peerj.7759)
Supplement: Data S2 [file peerj-07-7759-s002.docx]

**Sup. 2 A.** A549 and A427 cells (%) with AnnV+/PI- and AnnV+/PI+ staining

| A549 | **CTR** | **ICRP** | | |
| --- | --- | --- | --- | --- |
|  |  | **1.25** | **1.5** | **1.75** |
| **Experiment 1** | 5.6 | 16.7 | 50.4 | 78.5 |
|  | 5.0 | 12.3 | 48.0 | 79.2 |
|  | 4.9 | 14.8 | 53.0 | 78.9 |
| **Experiment 2** | 3.2 | 5.7 | 40.6 | 53.0 |
|  | 2.8 | 6.8 | 43.0 | 50.5 |
|  | 5.8 | 6.5 | 41.1 | 51.8 |
| **Experiment 3** | 5.7 | 9.2 | 50.1 | 54.0 |
|  | 5.7 | 5.3 | 50.3 | 56.0 |
|  | 2.6 | 16.3 | 39.6 | 55.0 |
| **MEAN** | **4.6** | **10.4** | **46.2** | **61.9** |
| **STD.DEV.** | **1.3** | **4.7** | **5.1** | **12.8** |

| A427 | **CTR** | **ICRP** | | |
| --- | --- | --- | --- | --- |
|  |  | **1.25** | **1.5** | **1.75** |
| **Experiment 1** | 6.4 | 25.5 | 42.5 | 86.0 |
|  | 6.2 | 28.9 | 47.5 | 88.5 |
|  | 6.3 | 32.2 | 45.3 | 87.0 |
| **Experiment 2** | 7.4 | 33.0 | 41.9 | 81.2 |
|  | 8.1 | 30.8 | 40.2 | 77.9 |
|  | 7.7 | 31.3 | 41.0 | 82.8 |
| **Experiment 3** | 5.2 | 5.2 | 53.6 | 67.0 |
|  | 3.2 | 7.8 | 51.7 | 70.0 |
|  | 4.2 | 6.6 | 52.8 | 68.5 |
| **MEAN** | **6.3** | **24.3** | **45.5** | **80.0** |
| **STD.DEV.** | **1.6** | **11.3** | **5.0** | **7.9** |

**Sup. 2 B.** APC-Annexin-V (FL4-H) and Propidium Iodide (FL2-H) staining representative dot plots


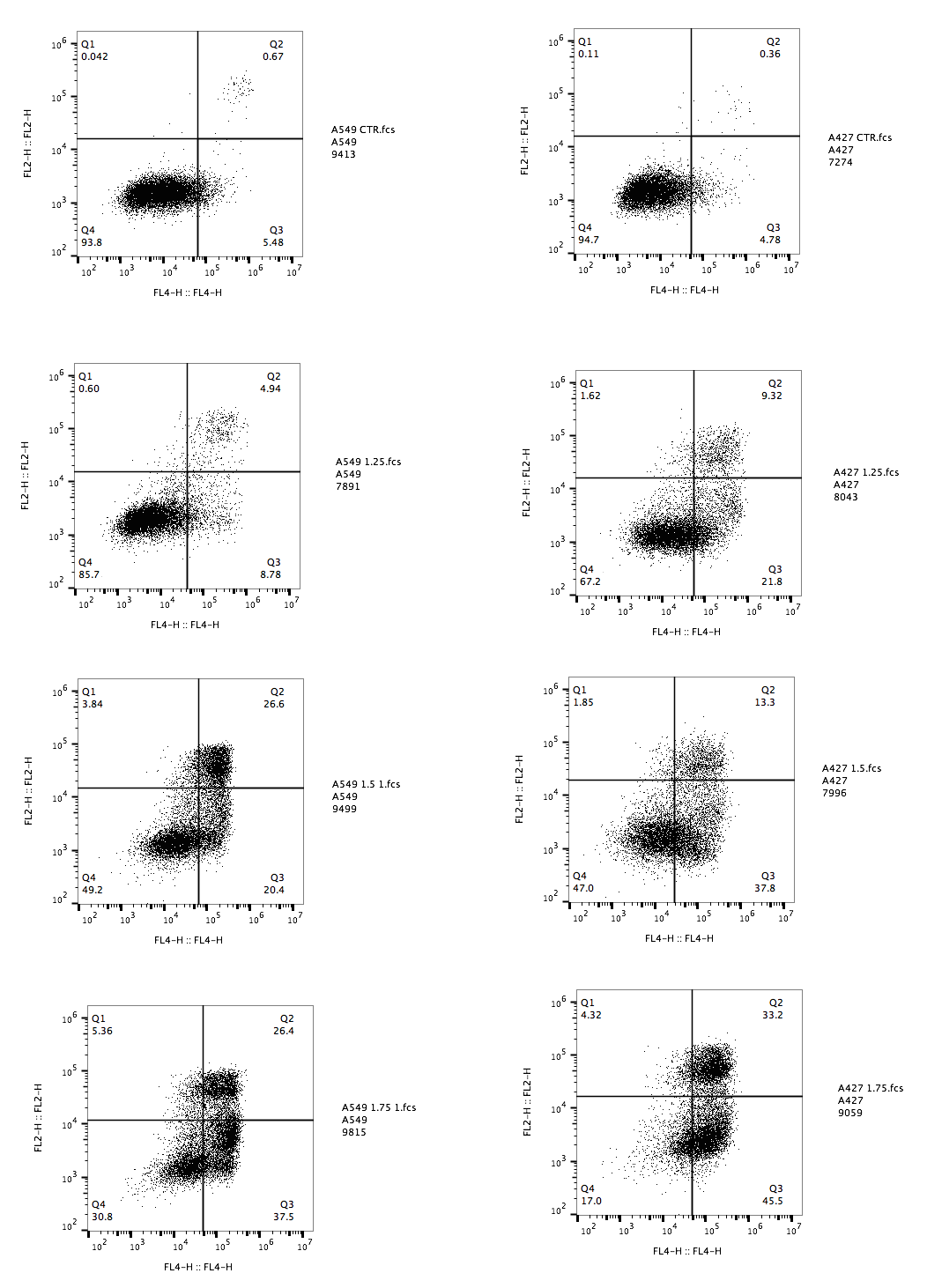


**Sup. 2 C**

**Clonogenic analyses**

| **A549** | # colonies | | | |
| --- | --- | --- | --- | --- |
|  | Control | ICRP | | |
|  |  | 1.0 U/mL | 1.25 U/mL | 1.5 U/mL |
| Exp. 1 | 45 | 8 | 2 | 0 |
|  | 44 | 10 | 1 | 1 |
|  | 47 | 9 | 0 | 1 |
| Exp. 2 | 53 | 6 | 6 | 3 |
|  | 47 | 7 | 2 | 2 |
|  | 40 | 5 | 5 | 6 |
| Exp. 3 | 41 | 8 | 3 | 0 |
|  | 51 | 10 | 0 | 0 |
|  | 48 | 5 | 5 | 5 |
| Mean | 46.2 | 7.6 | 2.7 | 2.0 |
| S.D. | 4.3 | 1.9 | 2.2 | 2.2 |
|  |  |  |  |  |
| Clonogenicity | **100%** | **3%** | **1%** | **1%** |
| S.D. | 9% | 1% | 2% | 2% |

| **A427** | # colonies | | | |
| --- | --- | --- | --- | --- |
|  | Control | ICRP | | |
|  |  | 1.0 U/mL | 1.25 U/mL | 1.5 U/mL |
| Exp. 1 | 49 | 18 | 4 | 3 |
|  | 57 | 36 | 0 | 1 |
|  | 52 | 7 | 0 | 1 |
| Exp. 2 | 45 | 15 | 1 | 2 |
|  | 36 | 10 | 0 | 0 |
|  | 41 | 11 | 5 | 0 |
| Exp. 3 | 50 | 5 | 0 | 1 |
|  | 38 | 29 | 5 | 1 |
|  | 55 | 14 | 0 | 2 |
| Mean | 47.0 | 16.1 | 1.7 | 1.2 |
| S.D. | 7.4 | 10.3 | 2.3 | 1.0 |
|  |  |  |  |  |
| Clonogenicity | **100%** | **7%** | **1%** | **1%** |
| S.D. | 16% | 1% | 3% | 2% |
