# Supplementary material for: Cytotoxic activity of IMMUNEPOTENT CRP against non-small cell lung cancer cell lines
Source: PeerJ. 2019 Sep 27;7:e7759. doi: 10.7717/peerj.7759 (PMC6768219; doi:10.7717/peerj.7759)
Supplement: Data S3 [file peerj-07-7759-s003.docx]

**Sup. 3A.** Cells (%) in different phases of cell cycle.

**A549 (Control)**

|  | **24h** | | | **48h** | | | **72h** | | |
| --- | --- | --- | --- | --- | --- | --- | --- | --- | --- |
|  | **G1** | **S** | **G2/M** | **G1** | **S** | **G2/M** | **G1** | **S** | **G2/M** |
| **Exp. 1** | 48.9 | 35.4 | 15.8 | 76.4 | 12.1 | 12.2 | 73.7 | 16.0 | 12.4 |
|  | 50.2 | 34.8 | 15.2 | 76.5 | 11.5 | 12.7 | 74.7 | 14.9 | 12.4 |
|  | 46.1 | 37.2 | 16.6 | 76.3 | 11.6 | 12.8 | 74.5 | 15.7 | 12.2 |
| **Mean** | 48.4 | 35.8 | 15.9 | 76.4 | 11.7 | 12.6 | 74.3 | 15.5 | 12.3 |
| **Std. Dev.** | 2.1 | 1.2 | 0.7 | 0.1 | 0.3 | 0.3 | 0.5 | 0.6 | 0.1 |
|  |  |  |  |  |  |  |  |  |  |
|  | **G1** | **S** | **G2/M** | **G1** | **S** | **G2/M** | **G1** | **S** | **G2/M** |
| **Exp. 2** | 51.9 | 32.3 | 15.7 | 76.3 | 12.6 | 13.0 | 75.5 | 14.0 | 12.7 |
|  | 56.7 | 27.1 | 16.1 | 76.4 | 13.0 | 12.7 | 75.7 | 13.1 | 13.0 |
|  | 57.8 | 26.7 | 15.4 | 76.3 | 11.5 | 13.7 | 76.8 | 12.1 | 12.6 |
| **Mean** | 55.5 | 28.7 | 15.7 | 76.3 | 12.4 | 13.1 | 76.0 | 13.1 | 12.8 |
| **Std. Dev.** | 3.1 | 3.1 | 0.4 | 0.1 | 0.8 | 0.5 | 0.7 | 1.0 | 0.2 |
|  |  |  |  |  |  |  |  |  |  |
|  | **G1** | **S** | **G2/M** | **G1** | **S** | **G2/M** | **G1** | **S** | **G2/M** |
| **Exp. 3** | 50.1 | 33.7 | 17.1 | 75.6 | 13.8 | 12.1 | 74.8 | 14.0 | 12.9 |
|  | 47.5 | 34.2 | 19.2 | 75.9 | 13.3 | 11.8 | 75.0 | 14.3 | 12.5 |
|  | 45.3 | 37.0 | 18.6 | 76.1 | 13.2 | 11.7 | 75.6 | 13.6 | 12.5 |
| **Mean** | 47.6 | 35.0 | 18.3 | 75.9 | 13.4 | 11.9 | 75.1 | 14.0 | 12.6 |
| **Std. Dev.** | 2.4 | 1.8 | 1.1 | 0.3 | 0.3 | 0.2 | 0.4 | 0.4 | 0.2 |
|  |  |  |  |  |  |  |  |  |  |
| **Mean** | **50.5** | **33.2** | **16.6** | **76.2** | **12.5** | **12.5** | **75.1** | **14.2** | **12.6** |
| **SD** | **4.4** | **3.9** | **1.4** | **0.3** | **0.9** | **0.6** | **0.9** | **1.2** | **0.3** |

**A549 (I-CRP)**

|  | **24h** | | | **48h** | | | **72h** | | |
| --- | --- | --- | --- | --- | --- | --- | --- | --- | --- |
|  | **G1** | **S** | **G2/M** | **G1** | **S** | **G2/M** | **G1** | **S** | **G2/M** |
| **Exp. 1** | 35.9 | 27.5 | 36.1 | 63.0 | 19.9 | 14.1 | 56.1 | 31.3 | 11.9 |
|  | 37.8 | 31.8 | 30.1 | 59.5 | 20.4 | 15.9 | 55.6 | 32.9 | 10.6 |
|  | 36.4 | 28.7 | 34.4 | 59.1 | 22.0 | 16.8 | 55.5 | 34.1 | 9.4 |
| Mean | 36.7 | 29.3 | 33.5 | 60.5 | 20.8 | 15.6 | 55.7 | 32.8 | 10.6 |
| Std. Dev. | 1.0 | 2.2 | 3.1 | 2.1 | 1.1 | 1.4 | 0.3 | 1.1 | 1.3 |
|  |  |  |  |  |  |  |  |  |  |
|  | **G1** | **S** | **G2/M** | **G1** | **S** | **G2/M** | **G1** | **S** | **G2/M** |
| **Exp. 2** | 35.8 | 23.6 | 39.6 | 52.1 | 30.1 | 17.5 | 56.6 | 27.0 | 17.7 |
|  | 38.1 | 24.2 | 37.2 | 53.3 | 29.1 | 17.1 | 56.4 | 25.1 | 19.9 |
|  | 38.3 | 27.5 | 34.1 | 55.8 | 24.4 | 18.1 | 56.8 | 29.7 | 14.6 |
| Mean | 37.4 | 25.1 | 37.0 | 53.7 | 27.9 | 17.6 | 56.6 | 27.3 | 17.4 |
| Std. Dev. | 1.4 | 2.1 | 2.8 | 1.9 | 3.0 | 0.5 | 0.2 | 2.3 | 2.7 |
|  |  |  |  |  |  |  |  |  |  |
|  | **G1** | **S** | **G2/M** | **G1** | **S** | **G2/M** | **G1** | **S** | **G2/M** |
| **Exp. 3** | 33.6 | 20.3 | 40.7 | 57.7 | 22.1 | 18.3 | 56.9 | 27.9 | 14.3 |
|  | 30.1 | 24.4 | 47.3 | 54.9 | 22.8 | 20.3 | 56.7 | 29.9 | 13.6 |
|  | 33.9 | 19.5 | 50.8 | 53.7 | 25.4 | 19.0 | 58.0 | 30.1 | 10.9 |
| Mean | 32.5 | 21.4 | 46.3 | 55.4 | 23.4 | 19.2 | 57.2 | 29.3 | 12.9 |
| Std. Dev. | 2.1 | 2.6 | 5.1 | 2.1 | 1.7 | 1.0 | 0.7 | 1.2 | 1.8 |
|  |  |  |  |  |  |  |  |  |  |
| **Mean** | **35.5** | **25.3** | **38.9** | **56.6** | **24.0** | **17.5** | **56.5** | **29.8** | **13.7** |
| **SD** | **2.7** | **4.0** | **6.6** | **3.5** | **3.6** | **1.8** | **0.8** | **2.8** | **3.4** |

**Representative histograms**

**24 h**

**
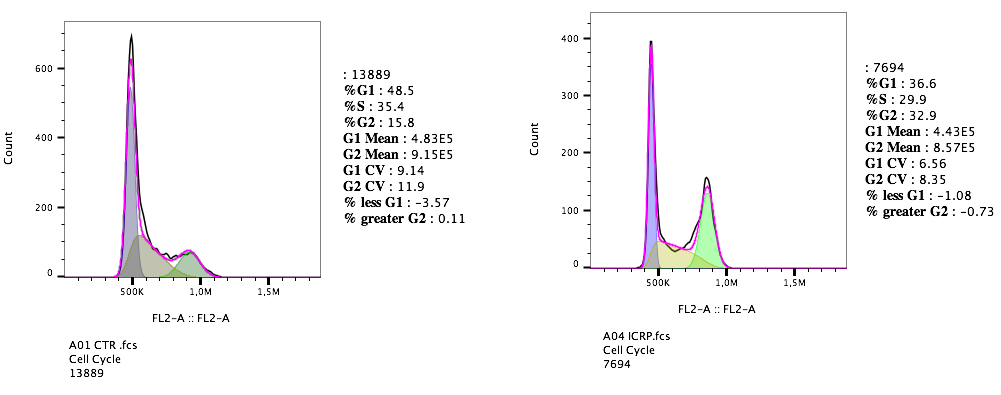
**

**48 h**

**
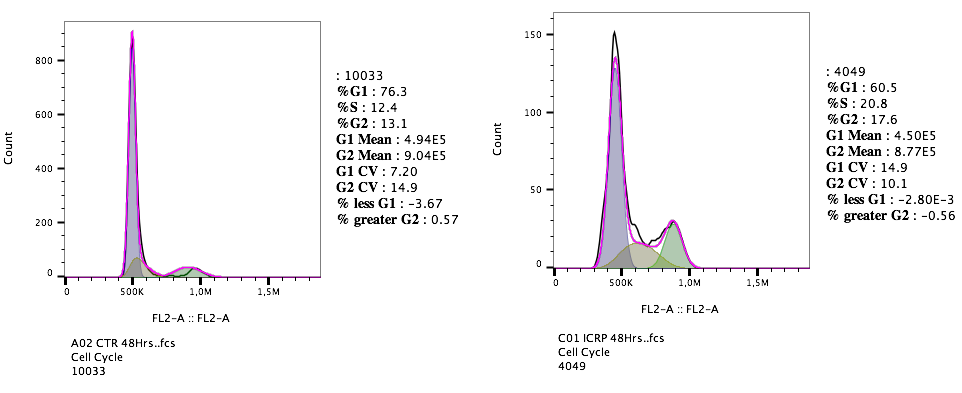
**

**72 h**

**
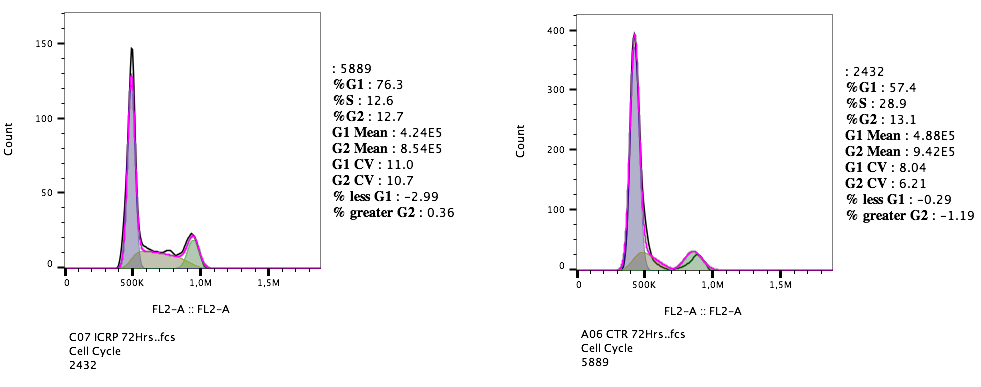
**

**A427 (Control)**

|  | **24h** | | | **48h** | | | **72h** | | |
| --- | --- | --- | --- | --- | --- | --- | --- | --- | --- |
|  | **G1** | **S** | **G2/M** | **G1** | **S** | **G2/M** | **G1** | **S** | **G2/M** |
| **Exp. 1** | 51.9 | 28.3 | 20.2 | 76.0 | 16.4 | 5.5 | 79.8 | 16.0 | 5.1 |
|  | 54.1 | 28.9 | 18.9 | 75.8 | 15.9 | 6.1 | 79.2 | 15.7 | 5.2 |
|  | 50.0 | 30.6 | 19.1 | 75.8 | 16.5 | 5.7 | 80.1 | 14.8 | 5.2 |
| Mean | 52.0 | 29.3 | 19.4 | 75.9 | 16.3 | 5.8 | 79.7 | 15.5 | 5.2 |
| Std. Dev. | 2.1 | 1.2 | 0.7 | 0.1 | 0.3 | 0.3 | 0.5 | 0.6 | 0.1 |
|  |  |  |  |  |  |  |  |  |  |
|  | **G1** | **S** | **G2/M** | **G1** | **S** | **G2/M** | **G1** | **S** | **G2/M** |
| **Exp. 2** | 67.3 | 25.5 | 8.7 | 78.5 | 15.9 | 5.4 | 82.1 | 12.1 | 6.0 |
|  | 61.1 | 31.7 | 8.6 | 78.6 | 14.3 | 5.3 | 81.9 | 13.4 | 5.6 |
|  | 64.0 | 27.5 | 9.4 | 78.4 | 15.2 | 6.2 | 83.2 | 11.5 | 5.8 |
| Mean | 64.1 | 28.2 | 8.9 | 78.5 | 15.1 | 5.6 | 82.4 | 12.3 | 5.8 |
| Std. Dev. | 3.1 | 3.2 | 0.4 | 0.1 | 0.8 | 0.5 | 0.7 | 1.0 | 0.2 |
|  |  |  |  |  |  |  |  |  |  |
|  | **G1** | **S** | **G2/M** | **G1** | **S** | **G2/M** | **G1** | **S** | **G2/M** |
| **Exp. 3** | 60.5 | 26.2 | 13.9 | 75.4 | 17.1 | 5.7 | 81.9 | 15.5 | 5.7 |
|  | 61.1 | 24.8 | 14.7 | 74.8 | 17.3 | 6.0 | 81.8 | 14.7 | 5.4 |
|  | 64.9 | 22.7 | 12.5 | 74.9 | 17.7 | 5.6 | 81.2 | 15.2 | 5.3 |
| Mean | 62.2 | 24.6 | 13.7 | 75.0 | 17.4 | 5.8 | 81.6 | 15.1 | 5.5 |
| Std. Dev. | 2.4 | 1.8 | 1.1 | 0.3 | 0.3 | 0.2 | 0.4 | 0.4 | 0.2 |
|  |  |  |  |  |  |  |  |  |  |
| **Mean** | **59.4** | **27.4** | **14.0** | **76.5** | **16.3** | **5.7** | **81.2** | **14.3** | **5.5** |
| **SD** | **6.1** | **2.9** | **4.6** | **1.6** | **1.1** | **0.3** | **1.3** | **1.6** | **0.3** |

**A427 (I-CRP)**

| **I-CRP** | **24h** | | | **48h** | | | **72h** | | |
| --- | --- | --- | --- | --- | --- | --- | --- | --- | --- |
|  | **G1** | **S** | **G2/M** | **G1** | **S** | **G2/M** | **G1** | **S** | **G2/M** |
| **Exp. 1** | 42.6 | 30.9 | 27.9 | 71.9 | 22.4 | 5.4 | 60.4 | 32.6 | 4.7 |
|  | 40.7 | 34.9 | 22.0 | 69.2 | 22.9 | 7.9 | 59.8 | 32.0 | 5.9 |
|  | 42.1 | 31.4 | 26.6 | 67.8 | 24.5 | 7.6 | 59.9 | 30.5 | 7.3 |
| Mean | 41.8 | 32.4 | 25.5 | 69.6 | 23.3 | 7.0 | 60.0 | 31.7 | 6.0 |
| Std. Dev. | 1.0 | 2.2 | 3.1 | 2.1 | 1.1 | 1.4 | 0.3 | 1.1 | 1.3 |
|  |  |  |  |  |  |  |  |  |  |
|  | **G1** | **S** | **G2/M** | **G1** | **S** | **G2/M** | **G1** | **S** | **G2/M** |
| **Exp. 2** | 43.9 | 29.9 | 25.5 | 51.8 | 30.6 | 15.7 | 61.6 | 28.8 | 10.6 |
|  | 42.1 | 29.9 | 29.6 | 49.0 | 33.1 | 15.1 | 61.2 | 32.9 | 5.2 |
|  | 41.1 | 33.6 | 24.2 | 48.1 | 36.6 | 14.8 | 61.5 | 29.0 | 8.2 |
| Mean | 42.4 | 31.1 | 26.4 | 49.6 | 33.4 | 15.2 | 61.4 | 30.2 | 8.0 |
| Std. Dev. | 1.4 | 2.1 | 2.8 | 1.9 | 3.0 | 0.5 | 0.2 | 2.3 | 2.7 |
|  |  |  |  |  |  |  |  |  |  |
|  | **G1** | **S** | **G2/M** | **G1** | **S** | **G2/M** | **G1** | **S** | **G2/M** |
| **Exp. 3** | 30.7 | 24.4 | 46 | 54.7 | 25.9 | 17.7 | 71.4 | 20.2 | 8.9 |
|  | 34.6 | 23.1 | 38.9 | 56.7 | 26.6 | 16.6 | 71.9 | 21.9 | 7.2 |
|  | 34.2 | 19.4 | 48.9 | 52.7 | 29.2 | 15.7 | 72.8 | 22.6 | 5.4 |
| Mean | 33.2 | 22.3 | 44.6 | 54.7 | 27.2 | 16.7 | 72.0 | 21.6 | 7.2 |
| Std. Dev. | 2.1 | 2.6 | 5.1 | 2.0 | 1.7 | 1.0 | 0.7 | 1.2 | 1.8 |
|  |  |  |  |  |  |  |  |  |  |
| **Mean** | **39.1** | **28.6** | **32.2** | **58.0** | **28.0** | **12.9** | **64.5** | **27.8** | **7.0** |
| **SD** | **4.7** | **5.2** | **9.9** | **9.2** | **4.8** | **4.6** | **5.7** | **5.0** | **2.0** |

**Representative histograms**

**24 h**

**
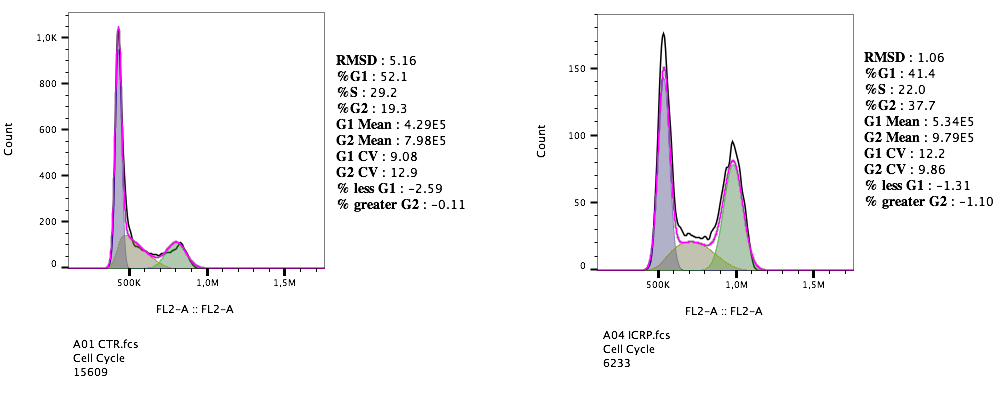
**

**48 h**

**
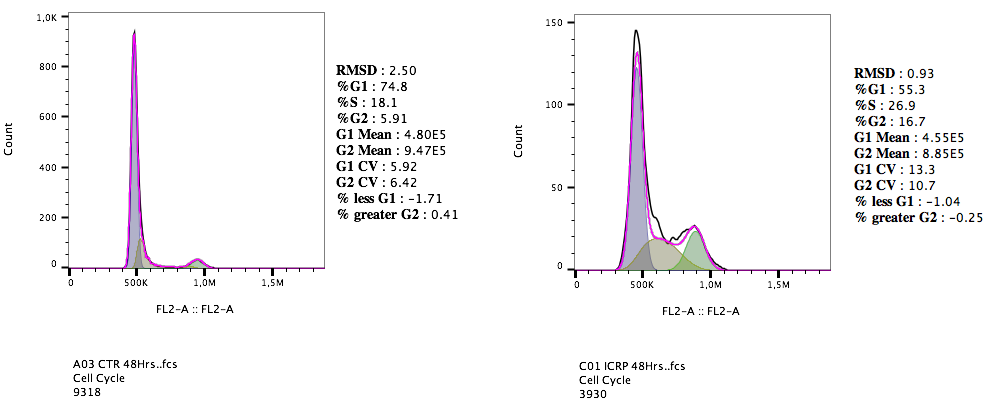
**

**72 h**

**
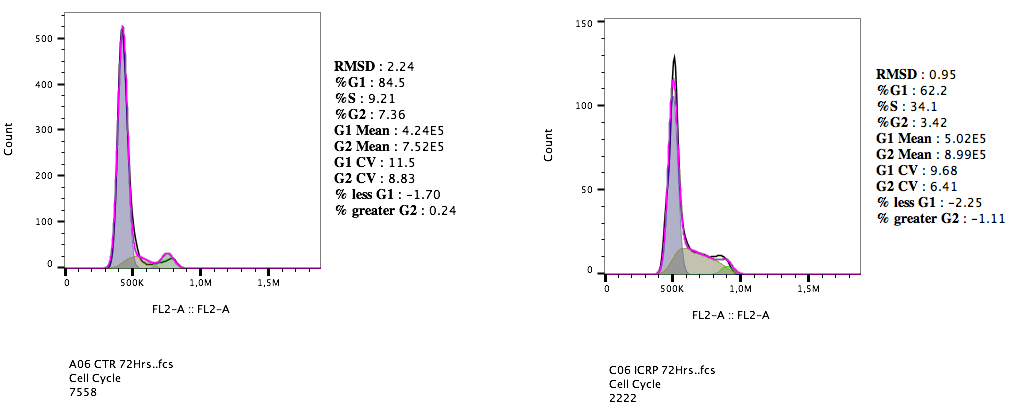
**
