# Supplementary material for: Cytotoxic activity of IMMUNEPOTENT CRP against non-small cell lung cancer cell lines
Source: PeerJ. 2019 Sep 27;7:e7759. doi: 10.7717/peerj.7759 (PMC6768219; doi:10.7717/peerj.7759)

**Sup. 4A. Cells (%) in sub-G1 phase**

| A549 | **CTR** | **24h** | **48h** | **72h** |
| --- | --- | --- | --- | --- |
| **Exp. 1** | 4.3 | 31.4 | 76.2 | 62.6 |
|  | 6.1 | 27.6 | 76.7 | 63.6 |
|  | 2.2 | 31.8 | 72.1 | 63.1 |
| **Exp. 2** | 1.9 | 31.6 | 58.8 | 62.1 |
|  | 1.7 | 33.1 | 48.2 | 62.2 |
|  | 1.7 | 32.4 | 50.8 | 62.0 |
| **Exp. 3** | 5.8 | 23.3 | 63.9 | 64.1 |
|  | 6.6 | 17.3 | 69.1 | 64.2 |
|  | 7.2 | 22.1 | 66.5 | 64.9 |
| **Mean** | **4.2** | **27.8** | **64.7** | **63.2** |
| **Std. Dev.** | **2.3** | **5.7** | **10.3** | **1.1** |

| A427 | **CTR** | **24h** | **48h** | **72h** |
| --- | --- | --- | --- | --- |
| **Exp. 1** | 10.1 | 20.9 | 75.4 | 85.2 |
|  | 12.8 | 16.6 | 76.9 | 84.9 |
|  | 12.4 | 12.2 | 51.2 | 84.6 |
| **Exp. 2** | 5.4 | 7.1 | 91.6 | 83.6 |
|  | 9.6 | 17.9 | 79.7 | 83.6 |
|  | 13.8 | 12.7 | 79.9 | 83.5 |
| **Exp. 3** | 14.1 | 27.5 | 67.1 | 84.4 |
|  | 12.0 | 25.5 | 66.3 | 83.8 |
|  | 17.0 | 30.0 | 60.1 | 83.0 |
| **Mean** | **11.9** | **18.9** | **72.0** | **84.1** |
| **Std. Dev.** | **3.3** | **7.7** | **12.1** | **0.7** |

**Sup. 4B. TMRE low cells (%)**

| A549 | **CTR** | **ICRP** |
| --- | --- | --- |
| **Experiment 1** | 4.6 | 71.7 |
|  | 4.4 | 76.2 |
|  | 3.2 | 74.0 |
| **Experiment 2** | 1.9 | 75.6 |
|  | 11.4 | 77.5 |
|  | 8.0 | 76.5 |
| **Experiment 3** | 10.8 | 67.6 |
|  | 15.3 | 68.0 |
|  | 13.1 | 67.8 |
| **Mean** | **8.1** | **72.8** |
| **STD.DEV.** | **4.7** | **4.1** |

| A427 | **CTR** | **ICRP** |
| --- | --- | --- |
| **Experiment 1** | 6.6 | 74.4 |
|  | 5.9 | 70.2 |
|  | 6.3 | 69.5 |
| **Experiment 2** | 12.3 | 70.1 |
|  | 11.4 | 65.9 |
|  | 11.8 | 62.9 |
| **Experiment 3** | 15.2 | 57.6 |
|  | 15.5 | 71.7 |
|  | 15.4 | 59.3 |
| **Mean** | **11.1** | **66.8** |
| **STD.DEV.** | **4.0** | **5.7** |

**Representative histograms’ gating for SubG1**


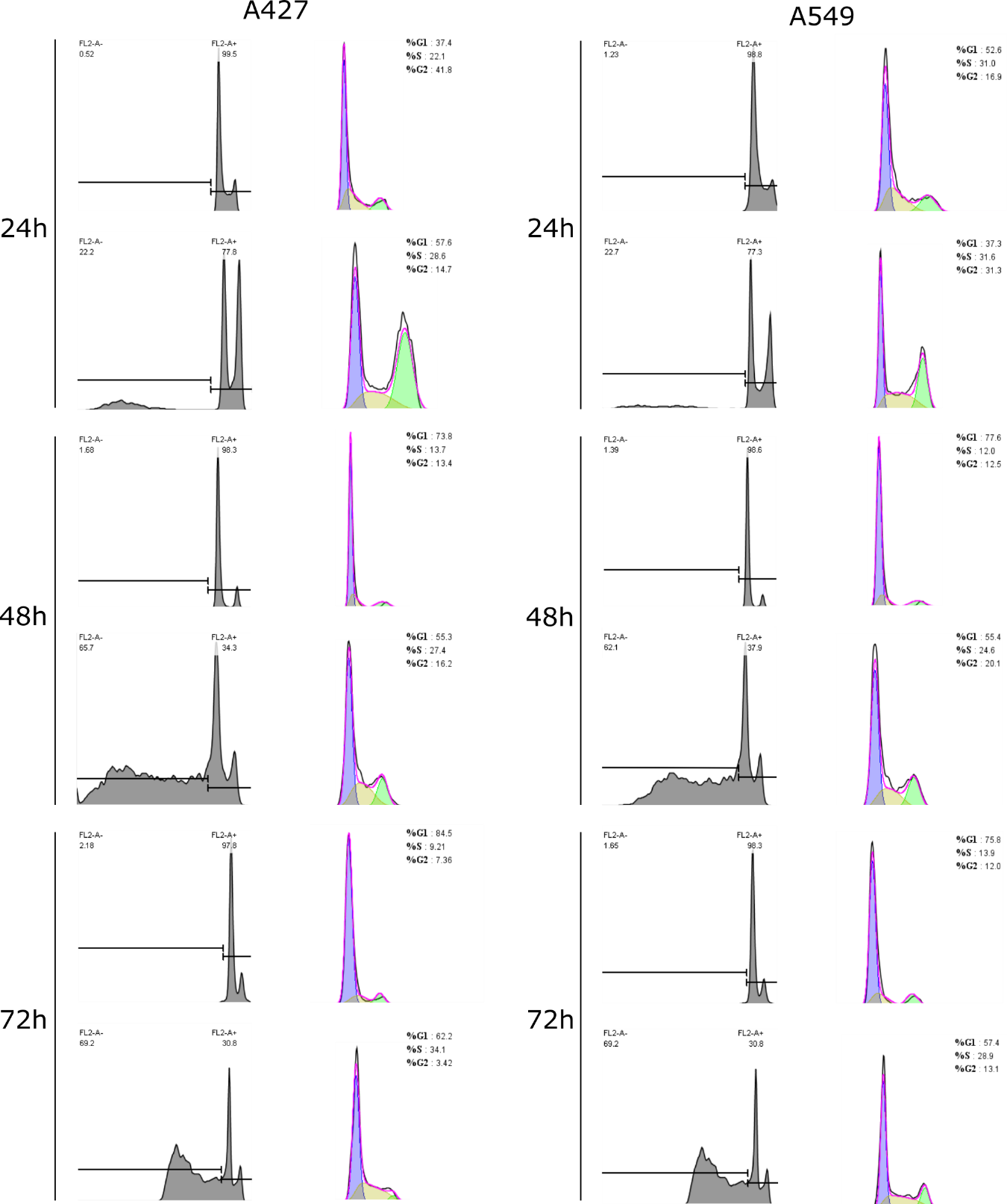


**Representative histograms For TMRE**

A549


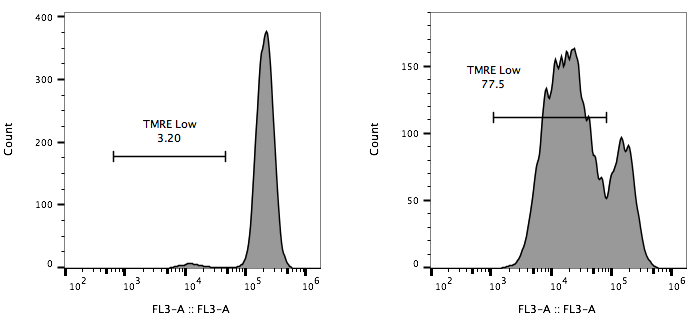


A549 TMRE CTR.fcs A549 TMRE 1.5.fcs

A549 A549

8564 12399

A427


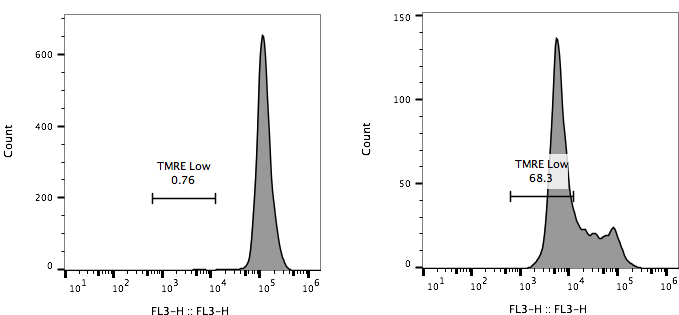


A427 TMRE CTR.fcs A427 TMRE 1.5.fcs

A427 A427

3723 11052

**Sup. 4C. Cell death by Ann/PI (%) in presence or absence of QVD**

| A549 | **CTR** | **ICRP** | **ETO** | **CTR + QVD** | **ICRP + QVD** | **ETO + QVD** |
| --- | --- | --- | --- | --- | --- | --- |
|  |  |  |  |  |  |  |
| **Experiment 1** | 5.6 | 50.4 | 58.0 | 6.1 | 70.0 | 4.2 |
|  | 5.0 | 48.0 | 60.1 | 3.5 | 68.0 | 8.4 |
|  | 4.9 | 53.0 | 56.5 | 4.8 | 69.0 | 6.3 |
| **Experiment 2** | 3.2 | 40.6 | 62.1 | 4.2 | 49.0 | 7.2 |
|  | 2.8 | 43.0 | 64.1 | 4.9 | 46.0 | 5.9 |
|  | 5.8 | 41.1 | 60.6 | 5.5 | 43.0 | 4.5 |
| **Experiment 3** | 5.7 | 50.1 | 60.5 | 5.8 | 69.2 | 5.5 |
|  | 5.7 | 50.3 | 65.8 | 4.2 | 44.4 | 4.9 |
|  | 2.6 | 39.6 | 50.1 | 3.9 | 58.5 | 8.3 |
| **MEAN** | **4.6** | **46.2** | **59.8** | **4.8** | **57.5** | **6.1** |
| **STD.DEV.** | 1.3 | 5.1 | 4.6 | 0.9 | 11.8 | 1.6 |

| **A427** | **CTR** | **ICRP** | **ETO** | **CTR + QVD** | **ICRP + QVD** | **ETO + QVD** |
| --- | --- | --- | --- | --- | --- | --- |
|  |  |  |  |  |  |  |
| **Experiment 1** | 6.4 | 42.5 | 51.0 | 5.7 | 57.5 | 3.5 |
|  | 6.2 | 47.5 | 51.4 | 6.1 | 55.4 | 4.1 |
|  | 6.3 | 45.3 | 50.6 | 5.5 | 59.8 | 3.0 |
| **Experiment 2** | 7.4 | 41.9 | 50.4 | 4.5 | 60.7 | 3.5 |
|  | 8.1 | 40.2 | 49.9 | 4.1 | 52.3 | 3.5 |
|  | 7.7 | 41.0 | 50.9 | 5.0 | 66.9 | 3.0 |
| **Experiment 3** | 5.2 | 53.6 | 50.0 | 5.1 | 49.0 | 4.0 |
|  | 3.2 | 51.7 | 50.1 | 4.6 | 43.6 | 4.1 |
|  | 4.2 | 52.8 | 50.4 | 5.4 | 37.8 | 2.5 |
| **MEAN** | **6.3** | **45.5** | **50.5** | **5.1** | **53.7** | **3.5** |
| **STD.DEV.** | 1.6 | 5.0 | 0.5 | 0.6 | 9.1 | 0.6 |

**Representative histograms**


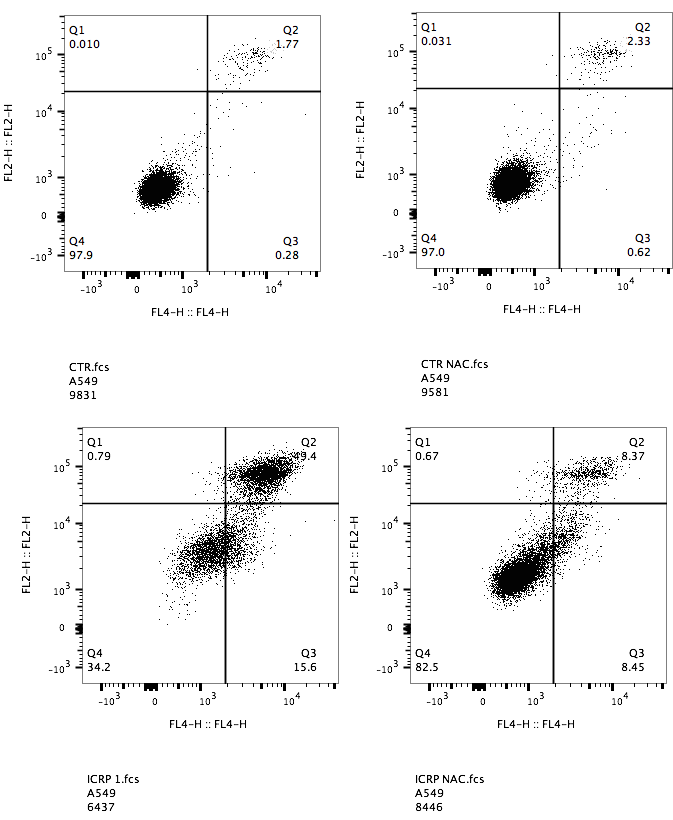

Supplement: Data S4 [file peerj-07-7759-s004.docx]
