# Supplementary material for: Cytotoxic activity of IMMUNEPOTENT CRP against non-small cell lung cancer cell lines
Source: PeerJ. 2019 Sep 27;7:e7759. doi: 10.7717/peerj.7759 (PMC6768219; doi:10.7717/peerj.7759)
Supplement: Figure S1 [file peerj-07-7759-s005.docx]

**Sup. 5A. I-CRP-induced ROS production**

|  | **CTR** | **NAC** | **ICRP** | **ICRP + NAC** |
| --- | --- | --- | --- | --- |
| **Experiment 1** | 4.4 | 3.8 | 34.2 | 11.2 |
|  | 3.5 | 3.1 | 33.2 | 1.0 |
|  | 4.1 | 4.8 | 33.7 | 2.6 |
| **Experiment 2** | 5.1 | 5.1 | 34.3 | 8.6 |
|  | 2.6 | 3.3 | 31.5 | 3.9 |
|  | 3.9 | 4.2 | 33.8 | 8.8 |
| **Experiment 3** | 6.9 | 3.4 | 36.8 | 0.2 |
|  | 7.5 | 3.6 | 36.7 | 1.1 |
|  | 6.4 | 3.4 | 36.8 | 0.9 |
| **Mean** | **4.9** | **3.9** | **34.6** | **4.3** |
| **Std. Dev.** | **1.7** | **0.7** | **1.8** | **4.2** |

**Representative histograms**


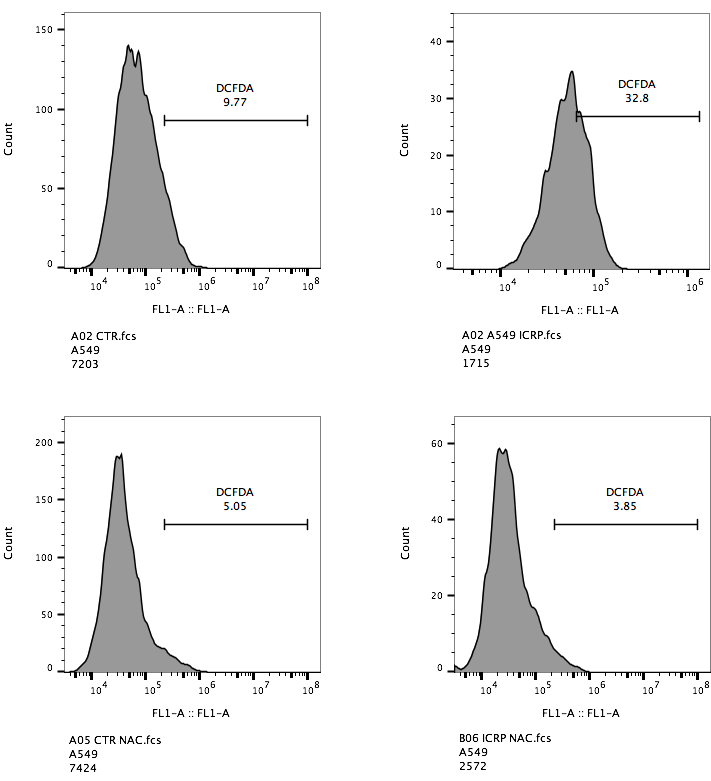


**Sup. 5B. ROS dependence on cell death**

| **AnnPI** | **CTR** | **NAC** | **ICRP** | **ICRP + NAC** |
| --- | --- | --- | --- | --- |
| **Experiment 1** | 1.9 | 3.1 | 61.9 | 10.2 |
|  | 1.7 | 3.5 | 62.6 | 20.0 |
|  | 1.8 | 3.4 | 63.0 | 11.5 |
| **Experiment 2** | 2.1 | 3.8 | 55.3 | 17.1 |
|  | 2.6 | 2.9 | 62.6 | 18.4 |
|  | 2.4 | 3.9 | 60.1 | 18.5 |
| **Experiment 3** | 2.4 | 2.2 | 31.4 | 13.2 |
|  | 2.1 | 3.0 | 35.0 | 9.9 |
|  | 1.5 | 1.8 | 31.3 | 8.5 |
| **Mean** | **2.1** | **3.1** | **51.5** | **14.1** |
| **Std. Dev.** | **0.4** | **0.7** | **14.4** | **4.4** |
